# Gut-to-Liver Escape of Viable *Streptococcus salivarius* Fuels Alcoholic Liver Disease via Fn14-Dependent NF-κB Signaling

**DOI:** 10.3390/microorganisms14102297

**Published:** 2026-10-09

**Authors:** Ranran Zhao, Xinqian Gao, Shuaiyi Liang, Jiegang Huang, Zuming Ruan, Zongmao Li, Guangkun Zhang, Ping Cui, Li Ye, Hui Chen

**Affiliations:** 1 School of Public Health, Guangxi Medical University, Nanning 530021, China; 202410200@sr.gxmu.edu.cn (R.Z.); jieganghuang@gxmu.edu.cn (J.H.); 2 Guangxi Key Laboratory of AIDS Prevention and Treatment, Guangxi Medical University, Nanning 530021, China; 202521895@sr.gxmu.edu.cn (X.G.); 202421822@sr.gxmu.edu.cn (G.Z.); 3 Life Sciences Institute, Guangxi Medical University, Nanning 530021, China; 202510282@sr.gxmu.edu.cn; 4 Collaborative Innovation Centre of Regenerative Medicine and Medical BioResource Development and Application Co-Constructed by the Province and Ministry, Nanning 530021, China; 5 Department of Gastroenterology, Minzu Hospital of Guangxi Zhuang Autonomous Region, Nanning 530001, China; rzm1220@163.com; 6 Department of Geriatric Gastroenterology, The First Affiliated Hospital of Guangxi Medical University, Nanning 530021, China; 13977040825@163.com

**Keywords:** alcoholic liver disease, *Streptococcus salivarius*, gut–liver axis, Fn14, NF-κB signaling pathway

## Abstract

Gut microbial dysbiosis contributes to alcoholic liver disease (ALD), but the pathogenic bacteria and mechanisms remain unclear. We investigated the role of *Streptococcus salivarius* (*S. salivarius*) in ALD mice using a modified 12-week NIAAA chronic-plus-binge ethanol regimen with oral gavage. *S. salivarius* aggravated hepatic steatosis, inflammation, fibrosis, and serum ALT elevation, while impairing colonic mucus and tight-junction integrity. Viable bacteria translocated from the gut to the liver. Integrated hepatic metabolomics and transcriptomics revealed disturbances in bile acid, glycerophospholipid, and choline metabolism and upregulation of *Tnfrsf12a*, encoding Fn14. *S. salivarius* activated NF-κB signaling without altering TLR2 or TLR4 abundance. Fn14 silencing or NF-κB inhibition attenuated the inflammatory response in vitro. Thus, our findings suggest that *S. salivarius* may contribute to ALD progression by impairing the intestinal barrier, translocating to the liver, and activating Fn14-dependent NF-κB signaling. The gut–liver translocation pathway and Fn14/NF-κB axis may represent therapeutic targets for ALD.

## 1. Introduction

Alcoholic liver disease (ALD), a chronic hepatic pathology driven by chronic and excessive alcohol abuse, represents a major global contributor to liver-related mortality [1]. The clinical progression of ALD spans a wide spectrum of pathologies, ranging from simple steatosis and alcoholic hepatitis to progressive fibrosis, cirrhosis, and ultimately hepatocellular carcinoma. It is estimated that alcohol consumption caused approximately 2.6 million deaths worldwide in 2019 [2]. Despite the escalating disease burden driven by a growing drinking population, the therapeutic armamentarium against ALD—especially at the cirrhotic and malignant stages—remains severely restricted. Currently, abstinence stands as the sole effective strategy, and no targeted pharmacotherapies have successfully transitioned into clinically approved regimens, leaving patient prognosis dismal [3,4]. Therefore, systematically deciphering the fundamental pathogenic mechanisms underlying ALD progression and identifying key pathogenic microbes along with their downstream functional pathways is of critical clinical urgency.

Recently, the gut–liver axis has shed new light on the mechanistic underpinnings of ALD [5,6]. Intestinal dysbiosis compromises intestinal barrier function, allowing the translocation of bacteria or their metabolic byproducts to the liver, thereby igniting hepatic inflammatory cascades and propelling fibrotic progression [7]. Previous studies have established that ALD patients harbor a severely disrupted gut microbiota, wherein the aberrant enrichment of specific genera is tightly coupled with the severity of hepatic damage [8,9]. Therefore, pinpointing key gut-derived strains with pathogenic potential and charting their molecular regulatory networks in driving ALD have become paramount to fully deconstructing the disease’s etiology.

In our previous study, the genus *Streptococcus* was found to be enriched in the gut microbiota of patients with alcoholic cirrhosis and was correlated with clinical markers of liver injury, including AST and ALT [8]. Subsequently, a *Streptococcus* strain was isolated and purified from fecal samples of patients with alcoholic cirrhosis and identified as *Streptococcus salivarius* (*S. salivarius*) through biochemical profiling, morphological characterization, and genomic sequencing. Although, strictly speaking, *S. salivarius* may not be the unequivocally dominant *Streptococcus* species across the entire cohort (as the species-level composition of *Streptococcus* in the cohort was not comprehensively resolved by 16S rRNA sequencing), functional prediction and our preliminary data suggest a potential role for *S. salivarius* and its metabolites in the pathogenesis of ALD. Consequently, the present study was designed to systematically dissect the pathogenic contribution of *S. salivarius* to ALD and elucidate its downstream molecular mechanisms.

To achieve this, we used a modified chronic-plus-binge ethanol feeding model with concurrent *S. salivarius* administration. In vivo bacterial tracking, fluorescence in situ hybridization (FISH), and culture-based assays were used to determine whether viable *S. salivarius* translocates from the gut to the liver. We then evaluated its effects on intestinal barrier integrity and hepatic steatosis, inflammation, and fibrosis. Integrated hepatic transcriptomic and metabolomic analyses were performed to characterize *S. salivarius*-associated molecular alterations and identify candidate signaling pathways. We further assessed the functional involvement of Fn14-dependent NF-κB signaling using Fn14 knockdown and NF-κB inhibition in vitro. Through these approaches, we aimed to define the contribution of gut-to-liver translocation of viable *S. salivarius* to ALD progression and to clarify the role of Fn14-dependent NF-κB signaling in this process.

## 2. Materials and Methods

### 2.1. Bacterial Strain and Culture Conditions

The *S. salivarius* strain used in this study was a clinical isolate obtained from fecal samples of patients hospitalized with alcoholic liver cirrhosis at the First Affiliated Hospital of Guangxi Medical University. The clinical isolate was designated *S. salivarius* GXMU-ALD-01 and was identified by 16S rRNA gene PCR amplification, Sanger sequencing, and comparison with reference sequences in the NCBI BLAST database. The closest matches were annotated as *S. salivarius* strain 4844 in the NCBI Core Nucleotide Database and *S. salivarius* strain ATCC 7073 in the NCBI 16S rRNA sequence database (Figure S1); these reference strains were used solely for sequence-based taxonomic identification and were not the clinical isolate used in this study. The 16S rRNA gene sequence of *S. salivarius* GXMU-ALD-01 has been deposited in the NCBI GenBank database, and the accession number is QB070364. The collection and use of patient fecal samples were approved by the Medical Ethics Committee of the First Affiliated Hospital of Guangxi Medical University (Approval No. 2021-KY-234). Written informed consent was obtained from all participants. *S. salivarius* was cultured in Brain Heart Infusion (BHI) broth at 37 °C with shaking at 200 rpm.

### 2.2. Mouse Model and Experimental Design

An ALD mouse model was established using the National Institute on Alcohol Abuse and Alcoholism (NIAAA) chronic-plus-binge ethanol feeding protocol with a Lieber-DeCarli liquid diet. Twelve-week-old female SPF C57BL/6 mice (n = 27 in total) were randomly assigned using a computer-generated random-number sequence to the control group (n = 6), model group (n = 10), or *S. salivarius* group (n = 11). No formal a priori power calculation was performed. The sample size was determined based on pilot experiments and previously published studies using comparable chronic-plus-binge ethanol models. Female mice were selected because of their higher susceptibility to alcohol-induced liver injury and their extensive use in previous experimental models of alcoholic liver disease.

After a 1-week acclimation period on a control liquid diet, the control group was fed a control liquid diet, while the model group and the *S. salivarius* group were fed a liquid diet containing 4% (*v*/*v*) ethanol. During the first 3 consecutive days, mice in the *S. salivarius* group were administered *S. salivarius* via oral gavage at a dose of 2 × 10^8^ CFU in 200 μL PBS per mouse daily, whereas the control and model groups received 200 μL of vehicle PBS. Thereafter, the respective oral gavages of *S. salivarius* or PBS were maintained at the same doses three times per week for a total duration of 12 weeks. Six hours prior to euthanasia, mice in the model and *S. salivarius* groups were subjected to a single acute gavage of 400 μL of a 31.5% (*w*/*v*) ethanol solution, while the control group received an isocaloric maltodextrin solution (400 μL).

Two mice in the model group died unexpectedly during the experimental period: one during the fifth week of the experiment and the other during the twelfth week of the experiment. The causes of death could not be determined. These mice were unavailable for subsequent tissue collection and were therefore excluded from all subsequent analyses. No other animals, samples, or data points were excluded. Consequently, the final numbers of animals available for analysis were n = 6, n = 8, and n = 11 in the control, model, and *S. salivarius* groups, respectively.

At the end of the experimental period, all mice were euthanized via cervical dislocation under excessive intraperitoneal anesthesia. Liver and intestinal tissues were rapidly harvested, and the isolated livers were weighed before being processed for subsequent analyses.

All experimental procedures were approved by the Animal Ethics Committee of Guangxi Medical University and were conducted in accordance with the Guide for the Care and Use of Laboratory Animals and internationally accepted principles for the care and use of laboratory animals.

### 2.3. Cell Culture, Transfection, and Treatment

HepG2 cells stably expressing CYP2E1 (HepG2-CYP2E1) were generated by lentiviral transduction, and LX2 cells were used as hepatic stellate cells. Both cell lines were cultured in Dulbecco’s Modified Eagle’s Medium (DMEM) supplemented with 10% fetal bovine serum (FBS), 100 U/mL penicillin, and 100 μg/mL streptomycin (1% Pen/Strep). Cells were maintained in a humidified incubator containing 5% CO_2_ and authenticated by short tandem repeat (STR) profiling. To establish an in vitro model of alcoholic liver injury, HepG2-CYP2E1 and LX2 cells were exposed to ethanol (EtOH) at concentrations of 100 mM and 50 mM, respectively, for 48 h.

For TNFRSF12A knockdown, HepG2-CYP2E1 and LX2 cells were transfected with siRNA targeting human *TNFRSF12A* (siTNFRSF12A; sense: 5′-CGCAGGAGAGAGAAGTTCA-3′) or a negative-control siRNA using riboFECT CP Reagent (Cat. No. C10511; Guangzhou RiboBio Co., Ltd., Guangzhou, China).

*S. salivarius* was cultured to the logarithmic growth phase, and the culture was centrifuged to collect the supernatant. The supernatant was passed through a 0.22-μm sterile filter to remove residual bacteria, yielding *S. salivarius* culture supernatant. HepG2-CYP2E1 cells were treated for 24 h with culture medium containing 10% *S. salivarius* culture supernatant.

Cell viability was evaluated using a CCK-8 assay to determine the effects of different multiplicities of infection (MOIs) of *S. salivarius* on cell viability. HepG2-CYP2E1 and LX2 cells were seeded in 96-well plates and cultured to an appropriate confluence. The cells were then co-cultured with logarithmic-phase *S. salivarius* at different MOIs in antibiotic-free medium for 4 h at 37 °C in a humidified incubator with 5% CO_2_. Control cells were treated with an equal volume of the corresponding bacteria-free medium. After co-culture, CCK-8 reagent was added according to the manufacturer’s instructions. Following incubation, absorbance was measured at 450 nm using a microplate reader. Cell viability was expressed as a percentage relative to the control group. To interrogate the involvement of Fn14-dependent NF-κB signaling, cells were allocated into five experimental sets: control, EtOH/ethanol-alone (Model), *S. salivarius* alone, and *S. salivarius* combined with either BAY 11–7082 or siTNFRSF12A. For NF-κB blockade, cells received BAY 11–7082 (10 μM; HY-13453, MedChemExpress, Monmouth Junction, NJ, USA) 1 h prior to a 4 h challenge with live *S. salivarius* at an MOI of 50:1. In parallel, Fn14-silenced cells were transfected with siTNFRSF12A and then subjected to the identical bacterial co-culture.

For the gentamicin protection assay, HepG2-CYP2E1 cells were co-cultured with logarithmic-phase *S. salivarius* at an MOI of 50:1 in antibiotic-free medium for 4 h at 37 °C in a humidified incubator with 5% CO_2_. After co-culture, the cells were washed with PBS to remove unbound bacteria and then incubated in culture medium containing gentamicin (100 μg/mL) for 2 h to eliminate extracellular bacteria. The cells were washed again with PBS and lysed. The cell lysates were serially diluted, plated onto BHI agar plates, and incubated for colony-forming unit (CFU) enumeration.

### 2.4. Lysine-Cy5.5 Labeling of Bacteria and In Vivo Fluorescence Tracing

To label *S. salivarius* for in vivo tracking, bacterial cultures in the logarithmic growth phase were incubated in BHI medium containing 10 μM Lysine-Cy5.5 (Q-Y082238, Xi’an Ruixi Biology, Xi’an, China) for 48 h. Successful conjugation of Lysine-Cy5.5 to the bacteria was confirmed by fluorescence microscopy. The Lysine-Cy5.5-labeled bacteria were then administered to mice by oral gavage. In vivo fluorescence imaging was performed using a live animal imaging system (AniView600, BLT, Guangzhou, China) before bacterial gavage and 18 h after gavage. After euthanasia, fluorescence signals in the liver were further examined.

### 2.5. Viable Bacterial Culture from Fresh Liver Tissues

Fresh mouse liver tissues were collected and homogenized in sterile tubes containing BHI broth. A total of 100 μL of tissue homogenate was evenly spread onto BHI agar plates using a sterile spreader and incubated in an anaerobic incubator at 37 °C for 48 h. To further isolate and purify potential *S. salivarius* colonies, colonies grown on BHI agar plates were transferred into BHI broth, thoroughly mixed, and subsequently plated onto Mitis Salivarius (M-S) agar, a selective differential medium. The plates were then incubated under anaerobic conditions at 37 °C for 48 h.

### 2.6. Fluorescence In Situ Hybridization Assay

Fluorescence in situ hybridization (FISH) was performed to detect *S. salivarius* in paraffin-embedded liver tissue sections using a FITC-labeled *S. salivarius*-specific probe (FISH probe: 5′-GCCCCTTTCAAATAAATGACATGTGT-3′, FITC-labeled). Hybridization buffer containing the FISH probe was applied to the tissue sections, followed by overnight incubation at 37–45 °C under humidified and light-protected conditions.

Briefly, paraffin sections were routinely deparaffinized and rehydrated, followed by antigen retrieval in citrate buffer (pH 6.0) at 90 °C for 40 min. After natural cooling, the sections were circled with a hydrophobic barrier pen and treated with proteinase K (20 μg/mL) at 40 °C for 5 min. The sections were then rinsed with distilled water and washed three times with PBS for 5 min each. Subsequently, prehybridization solution was added, and the sections were incubated at 40 °C for 1 h. After removal of the prehybridization solution, hybridization solution containing the FITC-labeled probe was applied, and the sections were incubated overnight in a humidified chamber at 40 °C in the dark.

On the following day, the hybridization solution was discarded, and the sections were sequentially washed with 2× SSC at 40 °C for 10 min, 1× SSC at 40 °C twice for 5 min each, and 0.5× SSC at room temperature for 10 min. After washing, DAPI staining solution was added, and the sections were incubated in the dark for approximately 8–10 min. Finally, the sections were rinsed with PBS, mounted with an antifade mounting medium, and observed and imaged under a fluorescence microscope.

### 2.7. Histopathological Analysis

Mouse liver, ileum, and colon tissues were harvested and fixed in 4% paraformaldehyde for at least 24 h, followed by paraffin embedding and sectioning. Paraffin-embedded sections were subjected to hematoxylin and eosin (H&E), Alcian Blue, and Sirius Red staining. Frozen liver sections were prepared for Oil Red O staining to assess lipid accumulation. All stained sections were imaged using a digital slide scanner to obtain full-slice scans. Quantitative analysis of the images was performed using ImageJ software (version 1.8.0).

### 2.8. Western Blot Analysis

Total protein was extracted using RIPA lysis buffer containing protease and phosphatase inhibitors. Protein concentrations were quantified using a BCA assay. Equal amounts of protein were separated by SDS-PAGE and transferred to PVDF membranes. After blocking with 5% non-fat milk or BSA for 1 h at room temperature, membranes were incubated with primary antibodies overnight at 4 °C, followed by appropriate secondary antibodies for 1 h at room temperature. Protein bands were visualized using an Odyssey CLx imaging system (LI-COR Biosciences, Lincoln, NE, USA).

### 2.9. Quantitative Real-Time PCR (RT-qPCR)

Total RNA was extracted from tissues or cells using TRIzol™ Reagent (15596026, Thermo Fisher Scientific, Waltham, MA, USA). Complementary DNA (cDNA) was synthesized using a reverse transcription kit (Takara Bio, Shiga, Japan). Quantitative real-time PCR (qPCR) was performed on a QuantStudio™ 5 Plus Real-Time PCR System (Applied Biosystems, Thermo Fisher Scientific). The primer sequences used are listed in Supplementary Table S1. GAPDH was used as the internal reference gene, and the relative expression levels of target genes were calculated using the 2^−ΔΔCt^ method.

### 2.10. S. salivarius-Specific qPCR Analysis of Liver Tissues

Total DNA was extracted from frozen mouse liver tissues using the QIAamp DNA Mini Kit (Qiagen, Hilden, Germany, ID. 51304). Briefly, approximately 20 mg of liver tissue was incubated with Buffer ATL and lysozyme (final concentration, approximately 10 mg/mL) at 37 °C for 1 h. Proteinase K was subsequently added, followed by incubation at 56 °C until complete tissue lysis. After lysis, RNase A was added and incubated at room temperature for 2 min to remove RNA. The lysate was then treated with Buffer AL and absolute ethanol, transferred to a QIAamp Mini spin column, and washed sequentially with Buffer AW1 and Buffer AW2 according to the manufacturer’s instructions. DNA was finally eluted with Buffer AE.

Quantitative real-time PCR (qPCR) was performed using *S. salivarius*-specific primers to detect *S. salivarius* DNA in liver tissues. Primer sequences are listed in Supplementary Table S1. For absolute quantification, genomic DNA from *S. salivarius* was extracted using the Bacteria Genomic DNA Kit (Aidlab, Gdańsk, Poland, DN11), serially diluted, and used to generate a standard curve. The copy number of *S. salivarius* DNA in liver tissues was calculated based on the Ct values and standard curve and normalized to tissue weight. Detailed parameters of the standard curve are provided in Supplementary Table S2, and the standard curve is shown in Supplementary Figure S2.

### 2.11. Biochemical Assays and ELISA

Serum levels of aspartate aminotransferase (AST; Cat. No. C010-2-1), alanine aminotransferase (ALT; Cat. No. C009-2-1), triglycerides (TG; Cat. No. A111-1-1), and total cholesterol (TC; Cat. No. A110-1-1) in mice were determined using commercial kits (Nanjing Jiancheng Bioengineering Institute, Nanjing, China) according to the manufacturer’s protocols. In addition, AST (Cat. No. JL12187, Jianglai, Shanghai, China) and ALT (Cat. No. JL13663, Jianglai, Shanghai, China) levels in cell culture supernatants were measured using enzyme-linked immunosorbent assay (ELISA) kits.

### 2.12. Statistical Analysis

Statistical analyses were performed using SPSS 22.0. Normality and homogeneity of variance were assessed by the Shapiro–Wilk test and Brown–Forsythe test, respectively. For normally distributed data with equal variances, one-way ANOVA followed by Dunnett’s test and Student’s *t*-test were used. For normally distributed data with unequal variances, Welch’s ANOVA with Dunnett’s post hoc test was applied. For non-normally distributed data, the Kruskal–Wallis test with Dunn’s post hoc (Bonferroni-corrected) and the Mann–Whitney U test were employed. All tests were two-tailed, with significance set at * *p* < 0.05, ** *p* < 0.01, *** *p* < 0.001 and **** *p* < 0.0001. Figures were generated using GraphPad Prism 10.1.2.

## 3. Results

### 3.1. S. salivarius Translocates to the Liver and Promotes ALD Progression in Mice

**Figure 1:**
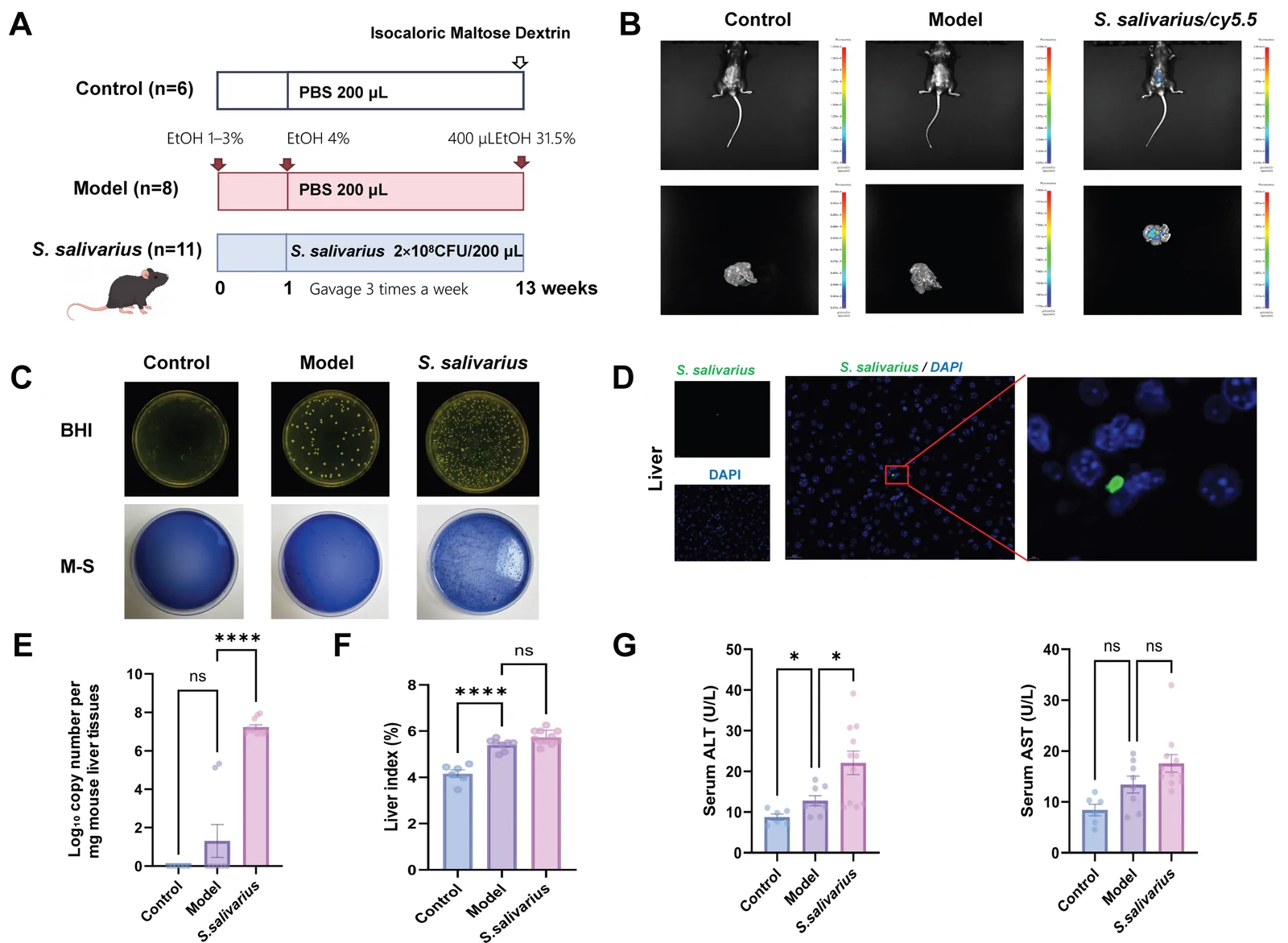
*S. salivarius* translocates to the liver and promotes disease progression in mice with alcoholic liver disease. (**A**). Experimental design for the alcoholic liver disease (ALD) mouse model, with Control (n = 6), Model (n = 8), and S. salivarius group (n = 11). (**B**) Representative in vivo fluorescence images showing the distribution of Cy5.5-lysine-labeled *S. salivarius* in mice at the experimental endpoint, followed by ex vivo fluorescence imaging of the excised liver tissues after euthanasia. (**C**) Culture of viable bacteria from liver tissue homogenates (upper panel, BHI medium; lower panel, M-S selective medium). (**D**) In situ hybridization of *S. salivarius* in mouse liver sections. *S. salivarius* signals are shown in green, and nuclei are counterstained with DAPI bule. Scale bar, 20 μm. (**E**) Absolute quantification of *S. salivarius* DNA in liver tissues by quantitative PCR (qPCR). The results are expressed as bacterial DNA copies per mg of liver tissue, based on a standard curve generated from serial dilutions of *S. salivarius* DNA. (**F**) Liver-to-body weight ratio (liver index) in the indicated groups. (**G**) Serum levels of alanine aminotransferase (ALT) and aspartate aminotransferase (AST). For quantitative analyses in panels (**E**–**G**), each data point represents one mouse. Data are presented as mean ± SEM. Control, n = 6; Model, n = 8; *S. salivarius*, n = 11. Statistical analysis was performed using one-way ANOVA with Dunnett’s test (**E**–**G**). * *p* < 0.05, **** *p* < 0.0001; ns, not significant.

To evaluate whether *S. salivarius* translocates to the liver and aggravates alcohol-induced liver injury, an ALD mouse model was established and mice were orally administered *S. salivarius* (Figure 1A). In vivo fluorescence imaging showed detectable Cy5.5-labeled *S. salivarius* signals in the abdomen and excised liver tissue (Figure 1B). Consistently, representative culture plates of liver homogenates showed no apparent bacterial colonies in the Control group, few colonies in the ALD model group, and visibly more colonies in the *S. salivarius* group (Figure 1C). FISH analysis further confirmed the presence of *S. salivarius* in liver sections (Figure 1D). Quantitative PCR using *S. salivarius*-specific primers further detected and quantified *S. salivarius* DNA in liver tissues from the *S. salivarius* group, whereas no amplification signal was detected in the Control group and in most samples from the Model group (Figure 1E). Furthermore, serum ALT levels in the *S. salivarius* group were significantly higher than those in the ALD model group, whereas serum AST levels showed an increasing trend without reaching statistical significance (Figure 1G; *p* < 0.05 and *p* > 0.05, respectively). Liver-to-body weight ratios (organ index) exhibited upward trends without reaching statistical significance (Figure 1F,G, *p* > 0.05). These findings indicate that viable *S. salivarius* can translocate to the liver and exacerbate ALD progression in mice.

### 3.2. S. salivarius Disrupts the Intestinal Barrier in ALD Mice

**Figure 2:**
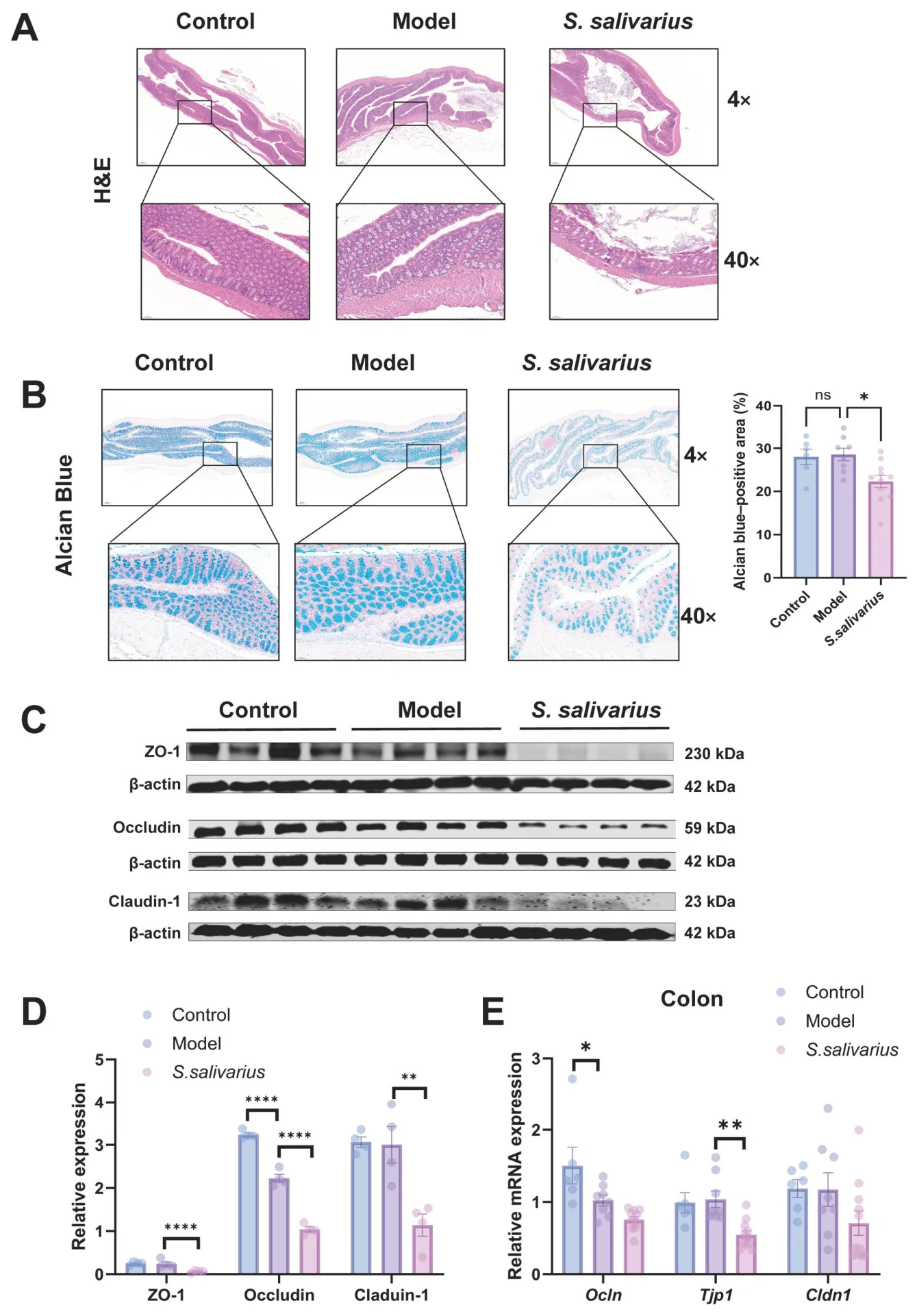
Effects of *S. salivarius* on intestinal permeability in mice with alcoholic liver disease. (**A**) Representative hematoxylin and eosin (H&E)-stained images of mouse colonic tissues. Scale bar, 50 μm. (**B**) Representative Alcian blue staining images and quantification of the Alcian blue-positive area in mouse colonic tissues. Scale bar, 50 μm. (**C**,**D**) Representative Western blot images and quantitative analysis of Occludin, ZO-1, and Claudin-1 protein expression in mouse colonic tissues. (**E**) Relative mRNA expression levels of the tight-junction genes *Ocln*, *Tjp1*, and *Cldn1* in mouse colonic tissues, as determined by quantitative reverse-transcription PCR (RT-qPCR). For quantitative analyses, each data point represents one mouse. Data are presented as mean ± SEM. Control, n = 6; Model, n = 8; *S. salivarius*, n = 11. Statistical analysis was performed using one-way ANOVA followed by Dunnett’s multiple-comparisons test, with the Model group as the reference group. * *p* < 0.05, ** *p* < 0.01, **** *p* < 0.0001; ns, not significant.

To investigate how *S. salivarius* translocates from the gut to the liver, we evaluated the integrity and function of the intestinal barrier in mice. In a qualitative assessment of H&E-stained colon tissues, the *S. salivarius* group appeared to show mucosal structural alterations and inflammatory cell infiltration in the lamina propria (Figure 2A). No quantitative histological scoring was performed. Alcian Blue (AB) staining further demonstrated a thinned colonic mucus layer and a significantly reduced AB-positive area in the *S. salivarius* group compared with the model group (Figure 2B, *p* < 0.05). Concurrently, Western blot analysis showed that the protein expression levels of the tight junction proteins ZO-1, Occludin, and Claudin-1 were significantly decreased in the colon tissues of the *S. salivarius* group (Figure 2C,D, *p* < 0.05). RT-qPCR analysis further confirmed that *S. salivarius* intervention significantly downregulated *Tjp1* mRNA expression in colon tissues (Figure 2E, *p* < 0.05). These findings indicate that *S. salivarius* can significantly damage colonic goblet cells and the mucus barrier structure, leading to impaired mucosal barrier function.

**Figure 3:**
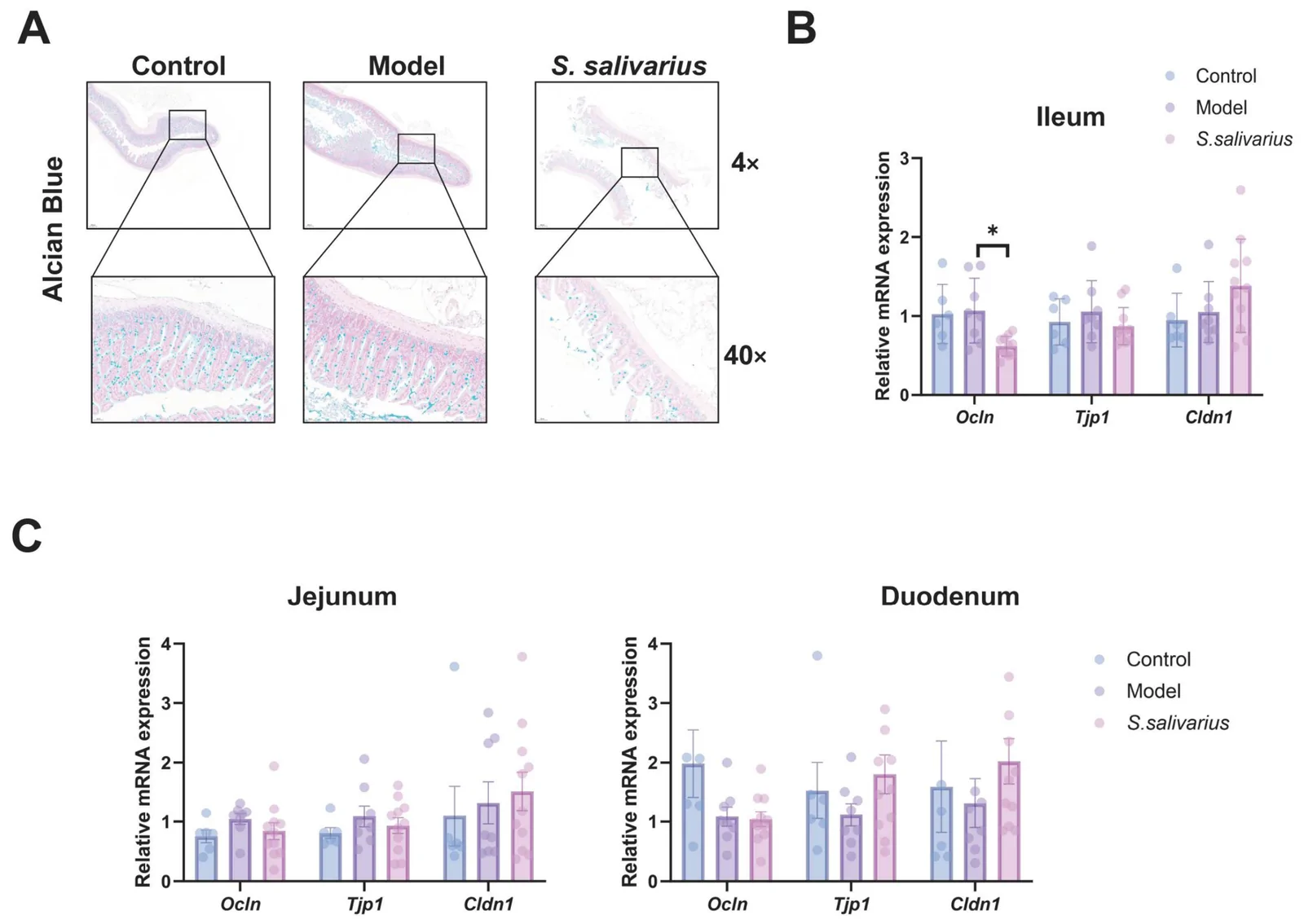
Effects of *S. salivarius* on intestinal permeability in mice with alcoholic liver disease. (**A**) Representative Alcian blue staining images of mouse ileal tissues. Scale bar: 50 μm. (**B**) Relative mRNA expression levels of the tight-junction genes *Ocln*, *Tjp1*, and *Cldn1* in mouse ileal tissues, as determined by quantitative reverse-transcription PCR (RT-qPCR). (**C**) Relative mRNA expression levels of *Ocln*, *Tjp1*, and *Cldn1* in mouse jejunal and duodenal tissues, as determined by RT-qPCR. For quantitative analyses in panels (**B**,**C**), each data point represents one mouse. Data are presented as mean ± SEM. Control, n = 6; Model, n = 8; *S. salivarius*, n = 11. Statistical analysis was performed using one-way ANOVA followed by Dunnett’s multiple-comparisons test, with the Model group as the reference group. * *p* < 0.05.

In the ileum, AB staining showed a significant reduction in the number of AB-positive goblet cells in the ileal mucosa of *S. salivarius*-treated mice (Figure 3A). Furthermore, the mRNA expression level of *Ocln* in ileal tissues was significantly downregulated (Figure 3B, *p* < 0.05). In contrast, no significant changes were observed in the expression of intestinal barrier-related genes in the other examined intestinal segments (Figure 3C, *p* > 0.05). Collectively, these results demonstrate that *S. salivarius* primarily disrupts the barrier function of the lower gastrointestinal tract, specifically the ileum and colon, which may represent a critical prerequisite for its translocation across the gut barrier into the liver.

### 3.3. S. salivarius Perturbs Hepatic Lipid Metabolism in ALD Mice

**Figure 4:**
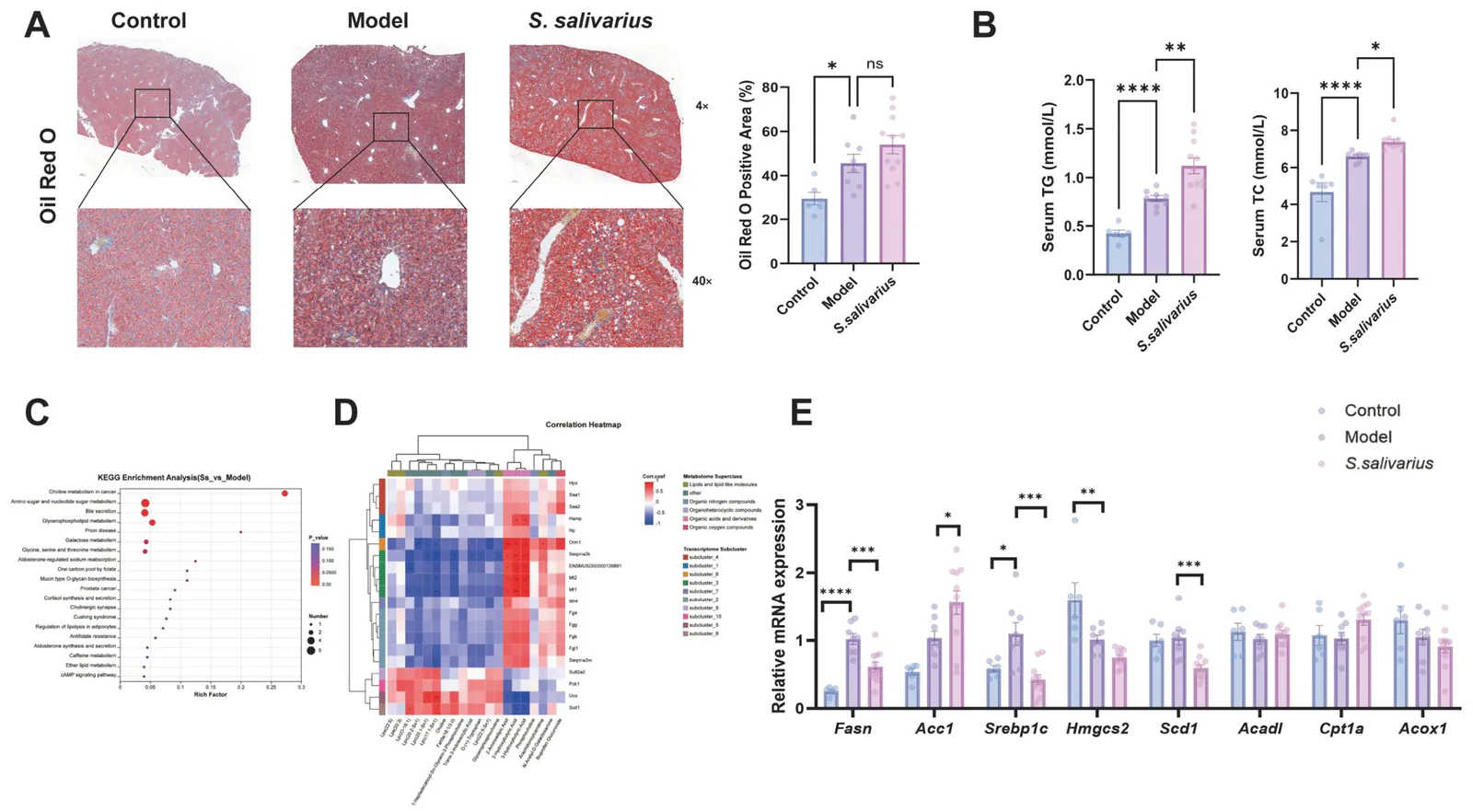
Effects of *S. salivarius* on hepatic lipid metabolism in mice with alcoholic liver disease. (**A**) Representative Oil Red O-stained images of mouse liver sections and quantification of the Oil Red O-positive area. Scale bar, 50 μm. (**B**) Serum triglyceride (TG) and total cholesterol (TC) levels. (**C**) Kyoto Encyclopedia of Genes and Genomes (KEGG) pathway enrichment analysis. (**D**) Heatmap of correlations between hepatic transcriptomic gene expression and metabolite classes. (**E**) Relative mRNA expression levels of lipid metabolism-related genes in mouse liver tissues, as determined by quantitative reverse-transcription PCR (RT-qPCR). For quantitative analyses in panels (**A**,**B**,**E**), each data point represents one mouse. Data are presented as mean ± SEM. Control, n = 6; Model, n = 8; *S. salivarius*, n = 11. Statistical analysis was performed using one-way ANOVA followed by Dunnett’s multiple-comparisons test, with the Model group as the reference group. * *p* < 0.05, ** *p* < 0.01, *** *p* < 0.001 and **** *p* < 0.0001; ns, not significant. Panels (**C**,**D**) show global analyses based on metabolomic data from six biological samples in the Model group and six biological samples in the *S. salivarius* group (Model, n = 6; *S. salivarius*, n = 6). Individual mouse-level data points are not represented in these panels.

To evaluate the specific impact of *S. salivarius* on lipid accumulation in ALD mice, Oil Red O staining of liver tissues was performed to assess lipid deposition, and serum TG and TC levels were measured. The *S. salivarius* group showed an upward trend in the Oil Red O-positive area compared with the Model group, although this difference did not reach statistical significance (Figure 4A, *p* > 0.05). Serum TG and TC levels were significantly elevated in the *S. salivarius* group compared with the ALD model group (Figure 4B, *p* < 0.05), indicating that *S. salivarius* exacerbates systemic lipid dysregulation in ALD mice.

To investigate the metabolic alterations, untargeted metabolomics analysis was performed on liver tissues from the Model and *S. salivarius* groups. Partial least squares discriminant analysis (PLS-DA) revealed clear separation between the two groups, indicating marked alterations in hepatic metabolic profiles following *S. salivarius* intervention (Figure S3A). A total of 111 upregulated and 83 downregulated metabolites were identified, with distinct distribution patterns shown by clustering analysis (Figure S3B,D). These metabolites were primarily classified as monosaccharides, steroid hormones, eicosanoids, fatty acids, carboxylic acids, and amino acids (Figure S3E). KEGG pathway enrichment analysis highlighted significant perturbations in multiple metabolic pathways, including choline metabolism in cancer, amino sugar and nucleotide sugar metabolism, bile secretion, and glycerophospholipid metabolism (Figure 4C). Integrated metabolomic–transcriptomic correlation analysis further showed that ketone body–related metabolites, including 3-hydroxybutyric acid and 2-hydroxybutyric acid, were positively associated with oxidative stress- and inflammation-related genes, whereas several lysophosphatidylcholine species were inversely correlated with these genes (Figure 4D). Collectively, these findings demonstrate that *S. salivarius* intervention markedly remodels the hepatic metabolic landscape in ALD mice, particularly affecting fatty acid oxidation and phospholipid metabolism in association with oxidative stress and inflammatory responses.

We measured the mRNA expression levels of key genes involved in hepatic lipogenesis (*Fasn*, *Acc1*, *Srebp1c*, *Hmgcs2*, *Scd1*) and lipolysis (*Acadl*, *Cpt1a*, *Acox1*) using RT-qPCR [10]. Interestingly, the expression of lipogenesis-related genes *Srebp1c*, *Fasn*, and *Scd1* was significantly downregulated in the *S. salivarius* group compared with the Model group, whereas the expression of *Acc1* was significantly upregulated (Figure 4E, *p* < 0.05). Collectively, these findings provide critical evidence that *S. salivarius* is actively involved in driving hepatic lipid metabolism disorders during ALD progression.

### 3.4. S. salivarius Promotes the Progression of Hepatic Inflammation and Fibrosis in ALD Mice

**Figure 5:**
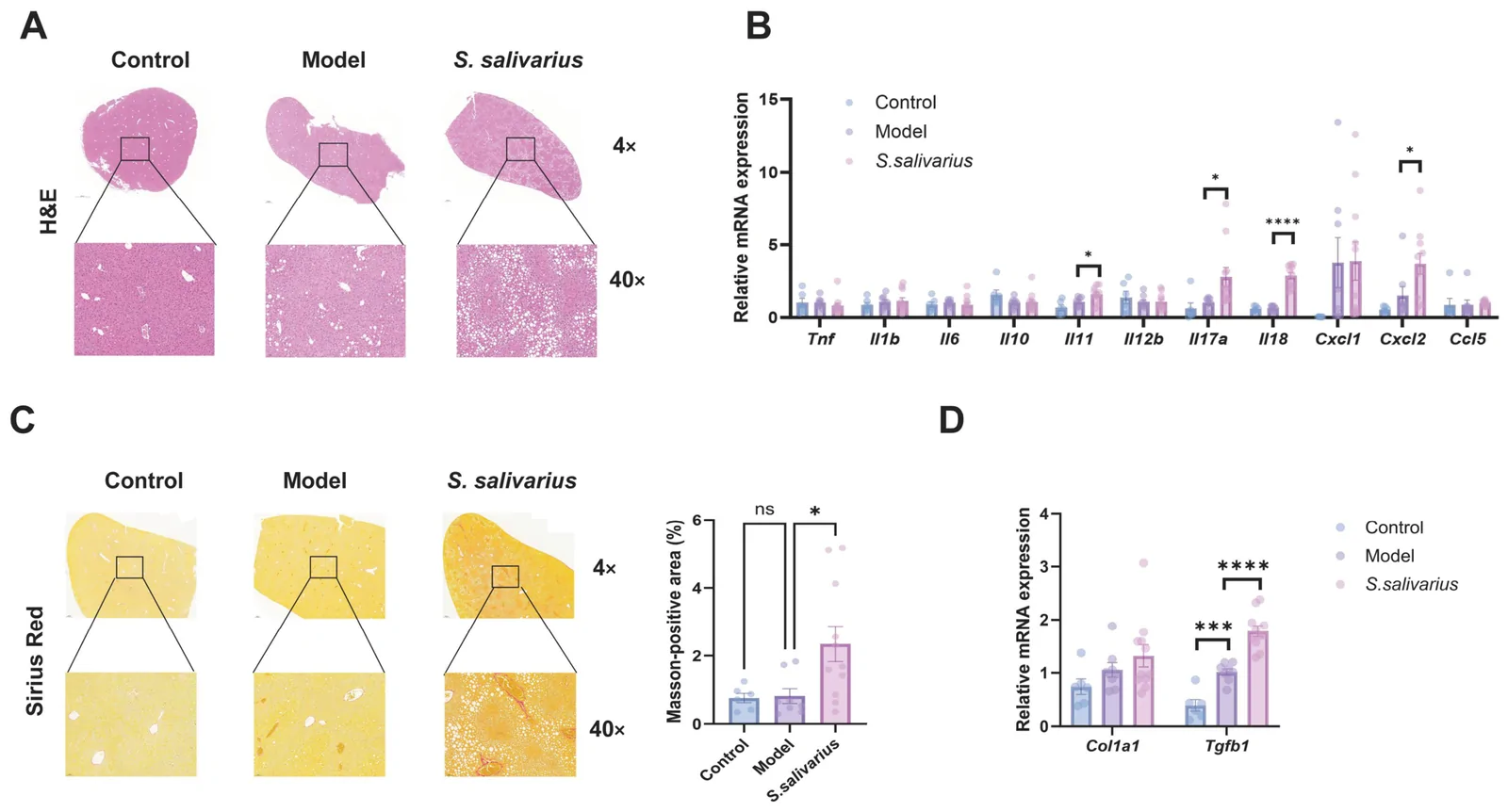
Effects of *S. salivarius* on hepatic inflammation and fibrosis in mice with alcoholic liver disease. (**A**) Representative hematoxylin and eosin (H&E)-stained images of mouse liver sections. Scale bar, 50 μm. (**B**) Relative mRNA expression levels of inflammation-related cytokine and chemokine genes in mouse liver tissues, as determined by quantitative reverse-transcription PCR (RT-qPCR). (**C**) Representative Sirius Red- stained images and quantification of the Sirius Red-positive area in mouse liver sections. Scale bar, 50 μm. (**D**) Relative mRNA expression levels of fibrosis-related genes in mouse liver tissues, as determined by RT-qPCR. For quantitative analyses in panels (**B**–**D**), each data point represents one mouse. Data are presented as mean ± SEM. Control, n = 6; Model, n = 8; *S. salivarius*, n = 11. Statistical analysis was performed using one-way ANOVA followed by Dunnett’s multiple-comparisons test, with the Model group as the reference group. * *p* < 0.05, *** *p* < 0.001 and **** *p* < 0.0001; ns, not significant.

To determine whether *S. salivarius* exacerbates hepatic injury in ALD, we performed H&E and Sirius Red staining of liver tissues from mice in different groups to evaluate pathological and fibrotic alterations. H&E staining revealed that *S. salivarius* intervention significantly aggravated pathological liver damage compared with the Model group. This damage was primarily characterized by pronounced hepatocellular steatosis, swelling, and ballooning degeneration of hepatocytes, accompanied by focal inflammatory cell infiltration (Figure 5A), demonstrating that *S. salivarius* exacerbates hepatic histopathological injury.

Furthermore, we assessed the mRNA expression levels of inflammatory cytokines, chemokines, and fibrosis-related genes in liver tissues. RT-qPCR analysis showed that the mRNA levels of interleukin-11 (*Il-11*), interleukin-17 (*Il17*), interleukin-18 (*Il18*), and the chemokine *Cxcl2* were significantly upregulated in the liver tissues of the *S. salivarius* group compared with the Model group (Figure 5B, *p* < 0.05). Additionally, Sirius Red staining demonstrated that the positive fibrotic area in the *S. salivarius* group was significantly larger than that in the ALD model group, indicating that liver fibrosis was further exacerbated and progressed beyond the baseline model level (Figure 5C). Consistently, the expression of the pivotal pro-fibrotic factor *Tgfb1* was also significantly elevated in the *S. salivarius* group (Figure 5D, *p* < 0.05). Taken together, these findings indicate that *S. salivarius* activates localized hepatic inflammatory responses and drives the progression of liver fibrosis.

### 3.5. S. salivarius Activates Hepatic NF-κB Signaling and Upregulates Fn14 in ALD Mice

**Figure 6:**
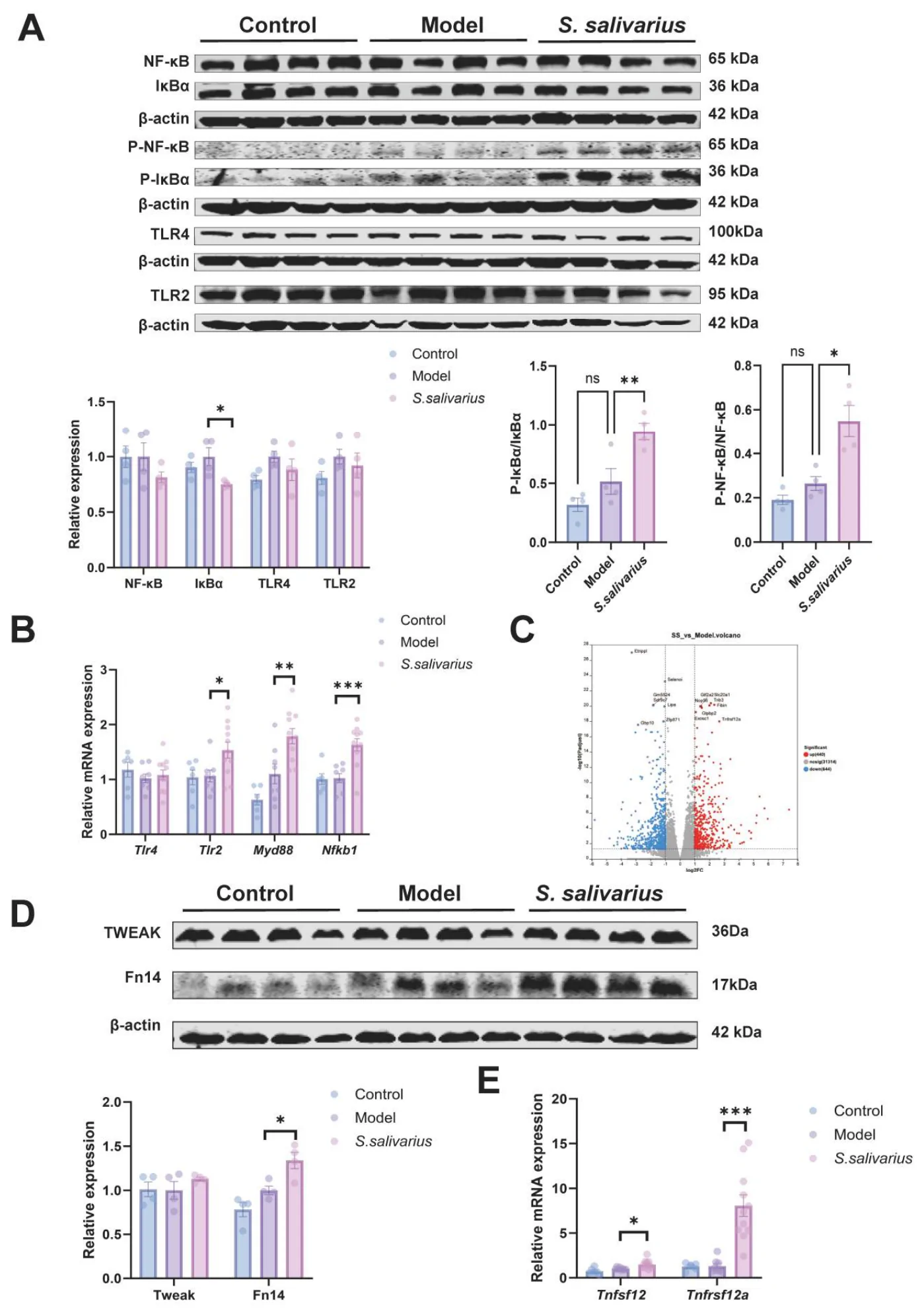
*S. salivarius* activates hepatic NF-κB signaling and upregulates Fn14 in ALD mice. (**A**) Representative Western blot images and quantitative analysis of TLR4, TLR2, MyD88, and NF-κB signaling pathway-related protein expression in mouse liver tissues. (**B**) Relative mRNA expression levels of *Tlr4*, *Myd88*, *Nfkb1*, and *Tlr2* in mouse liver tissues, as determined by quantitative reverse transcription PCR (RT-qPCR). (**C**) Volcano plot of differentially expressed genes between the Model group and the *S. salivarius* group. (**D**) Representative Western blot images and quantitative analysis of TWEAK and Fn14 protein expression in mouse liver tissues. (**E**) Relative mRNA expression levels of Tnfsf12 and Tnfrsf12a in mouse liver tissues, as determined by RT-qPCR. For quantitative analyses in panels (**A**,**B**,**D**,**E**), each data point represents one mouse. Data are presented as mean ± SEM. Control, n = 6; Model, n = 8; *S. salivarius*, n = 11. Statistical analysis was performed using one-way ANOVA followed by Dunnett’s multiple-comparisons test, with the Model group as the reference group. * *p* < 0.05, ** *p* < 0.01 and *** *p* < 0.001; ns, not significant. Panel (**C**) shows a global transcriptomic analysis based on six biological samples from the Model group and five biological samples from the *S. salivarius* group (Model, n = 6; *S. salivarius*, n = 5). Individual mouse-level data points are not represented in this panel.

To explore the molecular mechanisms by which *S. salivarius* promotes ALD progression, we assessed hepatic inflammatory signaling pathways. *S. salivarius* intervention significantly reduced IκBα expression and concurrently elevated phosphorylated p65 and *p*-IκBα levels, while total p65 remained unchanged (Figure 6A, *p* < 0.05), indicative of functional NF-κB activation in the liver. To determine whether this activation was mediated by canonical pathogen-recognition receptors, we assessed TLR2 and TLR4 expression. Although transcriptional upregulation of *Tlr2*, *Myd88*, and *Nfkb1* was observed in the *S. salivarius* group (Figure 6B, *p* < 0.05), their protein levels remained unchanged across all groups (Figure 6A, *p* > 0.05), suggesting that *S. salivarius* engages a non-canonical, receptor-independent route to activate hepatic NF-κB.

Principal component analysis (PCA) of the hepatic transcriptomic profiles showed a distinct clustering pattern between the Model and *S. salivarius* groups (Figure S4A). Differential expression analysis identified 440 upregulated and 644 downregulated genes (Figure 6C). KEGG enrichment analysis revealed that these differentially expressed genes were significantly involved in multiple pathways, including cell cycle, cell adhesion molecules, MAPK signaling, and metabolic processes such as fatty acid biosynthesis and elongation, as well as ferroptosis (Figure S4C). Notably, *Tnfrsf12a* (Log2FC = 2.67), encoding Fn14, was among the most significantly upregulated genes in the *S. salivarius* group. As the functional receptor for TWEAK [11], Fn14 can recruit TRAF2/5 to activate the IKK/ NF-κB signaling cascade. Further validation showed that *Tnfsf12* (encoding TWEAK) and *Tnfrsf12a* mRNA levels were significantly increased in the *S. salivarius* group, whereas Fn14 protein expression was elevated without a significant change in TWEAK protein levels (Figure 6D,E). These findings suggest that Fn14 may serve as a critical upstream mediator of *S. salivarius*-induced hepatic NF-κB activation.

### 3.6. Blockade of Fn14 and NF-κB Alleviates S. salivarius-Induced Hepatocyte Injury in HepG2 Cells

To assess the effects of soluble bacterial products, HepG2-CYP2E1 cells were treated with *S. salivarius* culture supernatant. The treatment did not significantly affect ALT or AST levels or the mRNA expression of inflammatory cytokines, including TNF and IL1B (Supplementary Figure S5), suggesting that the culture supernatant alone was insufficient to reproduce the hepatocellular injury and inflammatory responses induced by viable *S. salivarius*.

To further assess the interaction between viable *S. salivarius* and HepG2-CYP2E1 cells, we performed a gentamicin protection assay. After gentamicin treatment, the cells were lysed and plated, and a small number of viable bacteria remained detectable as colony-forming units (CFUs) (Supplementary Figure S6). These findings indicate an association between viable *S. salivarius* and HepG2-CYP2E1 cells, although the assay could not distinguish between tightly adherent and intracellular bacteria.

**Figure 7:**
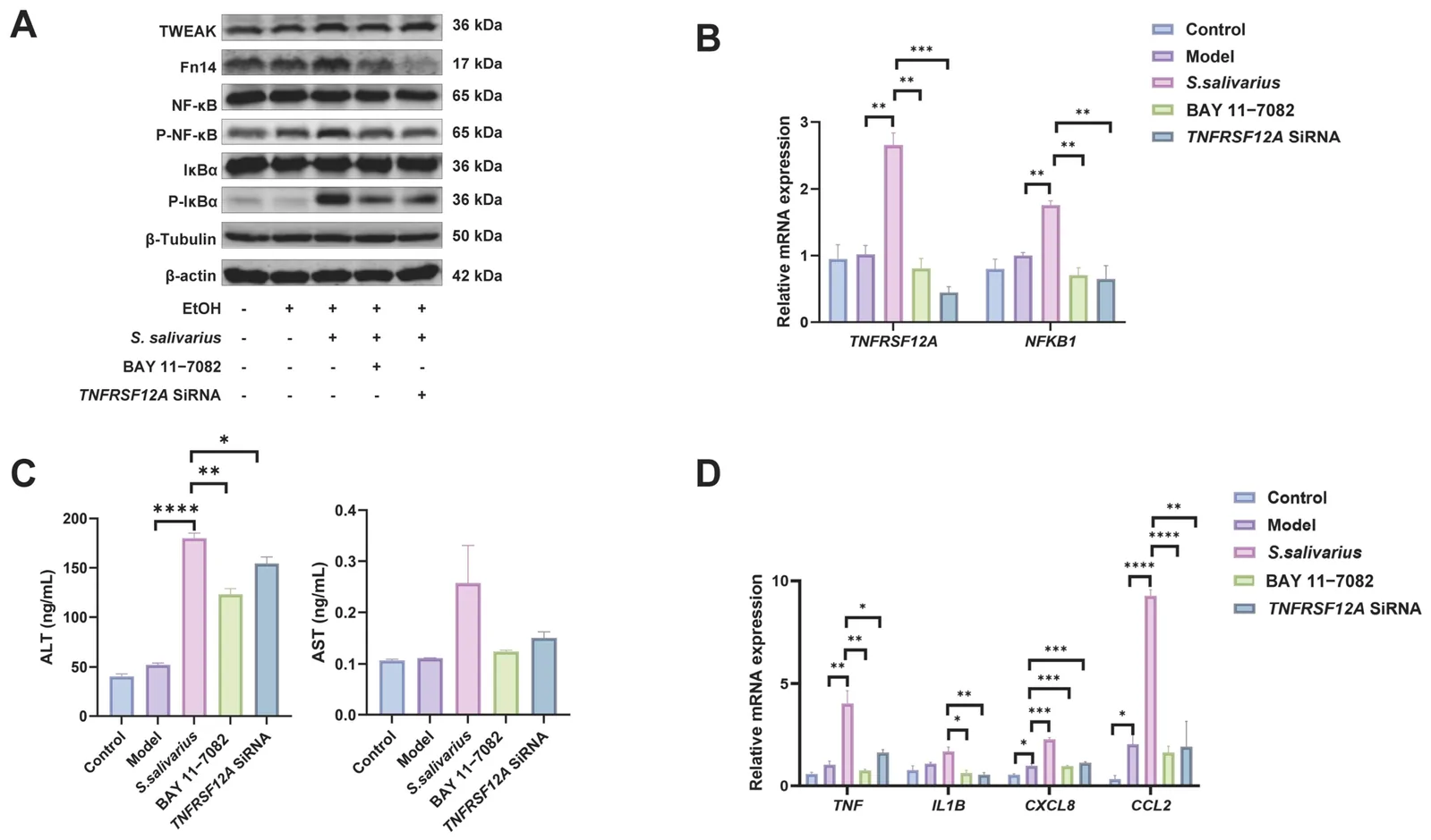
Blockade of Fn14 and NF-κB alleviates *S. salivarius*-induced hepatocyte injury in HepG2 cells (**A**) Representative Western blot images and quantitative analysis of proteins involved in the TWEAK/Fn14/NF-κB signaling pathway in HepG2-CYP2E1 cells. (**B**) Relative mRNA expression levels of *TNFRSF12A* and NFKB1 in HepG2-CYP2E1 cells, as determined by quantitative reverse-transcription PCR (RT-qPCR). (**C**) Alanine aminotransferase (ALT) and aspartate aminotransferase (AST) levels in the culture supernatants of HepG2-CYP2E1 cells. (**D**) Relative mRNA expression levels of inflammatory cytokines and chemokines in HepG2-CYP2E1 cells, as determined by RT-qPCR. Data are presented as mean ± SEM (n = 3). Statistical analysis was performed using one-way ANOVA followed by Dunnett’s multiple-comparisons test for comparisons among the Control, Model, and *S. salivarius* groups, with the Model group as the reference group. Comparisons between the *S. salivarius* group and the BAY 11–7082 or *TNFRSF12A* siRNA treatment groups were performed using an unpaired two-tailed Student’s *t*-test. * *p* < 0.05, ** *p* < 0.01, *** *p* < 0.001 and **** *p* < 0.0001.

To validate the role of *S. salivarius* in an in vitro model of alcoholic liver injury, we co-cultured logarithmic-phase *S. salivarius* with ethanol-treated HepG2-CYP2E1 cells at an MOI of 50:1 for 4 h. The viability of HepG2-CYP2E1 remained above 80% of the corresponding control levels, as determined by the CCK-8 assay (Supplementary Figure S7). Under these conditions, no significant differences were observed in the total protein levels of NF-κB and IκBα among the Control, Model, and *S. salivarius* groups (Figure 7A). However, the *S. salivarius* group exhibited significant upregulation of phosphorylated NF-κB (*p*-p65), *p*-IκBα, and Fn14 (the protein encoded by *TNFRSF12A*) (Figure 7A). Consistently, RT-qPCR revealed increased mRNA levels of *TNFRSF12A* and NFKB1 in the *S. salivarius* group compared with the Model group, and these increases were markedly attenuated by treatment with the NF-κB inhibitor BAY 11–7082 or siRNA-mediated knockdown of *TNFRSF12A* (Figure 7B, *p* < 0.05). To determine whether this pathway mediates *S. salivarius*-induced inflammation, we assessed pro-inflammatory cytokine expression and ALT release following pathway blockade. ELISA showed that *S. salivarius* significantly elevated ALT levels in the supernatant, an effect reversed by either *TNFRSF12A* siRNA or NF-κB inhibition (Figure 7C, *p* < 0.05). RT-qPCR further demonstrated that *S. salivarius* upregulated the mRNA levels of *TNF*, *CXCL8*, and *CCL2*, whereas Fn14 silencing or NF-κB inhibition significantly suppressed the expression of *TNF*, *IL1B*, *CXCL8*, and *CCL2* (Figure 7D, *p* < 0.05). Collectively, these findings indicate that *S. salivarius* provokes inflammatory responses in HepG2 cells through the Fn14/NF-κB axis, as genetic or pharmacological blockade of this pathway partially alleviates the pro-inflammatory effects.

### 3.7. Targeting Fn14 and NF-κB Signaling Alleviates S. salivarius-Induced Fibrotic Responses in LX2 Cells

**Figure 8:**
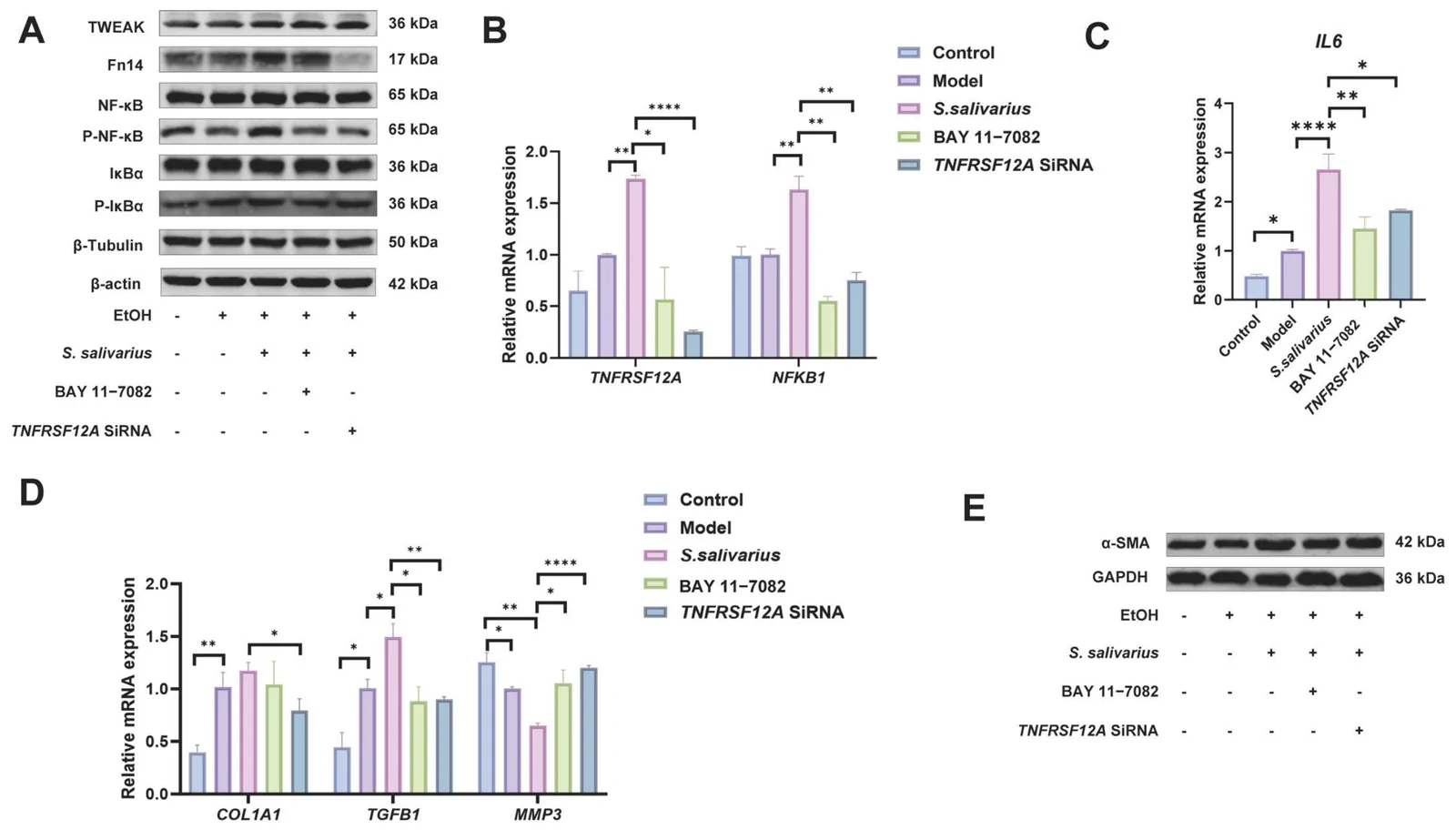
Targeting Fn14 and NF-κB signaling alleviates *S. salivarius*-induced fibrotic responses in LX2 cells. (**A**) Representative Western blot images of proteins involved in the TWEAK/Fn14/NF-κB signaling pathway in LX2 cells. (**B**) Relative mRNA expression levels of *TNFRSF12A* and *NFKB1* in LX2 cells, as determined by quantitative reverse-transcription PCR (RT-qPCR). (**C**) Relative mRNA expression levels of inflammatory factors in LX2 cells, as determined by RT-qPCR. (**D**) Relative mRNA expression levels of fibrosis-related factors in LX2 cells, as determined by RT-qPCR. (**E**) Representative Western blot images of α-smooth muscle actin (α-SMA) protein expression in LX2 cells. Data are presented as mean ± SEM (n = 3). Statistical analysis was performed using one-way ANOVA followed by Dunnett’s multiple-comparisons test for comparisons among the Control, Model, and *S. salivarius* groups, with the Model group as the reference group. Comparisons between the *S. salivarius* group and the BAY 11–7082 or *TNFRSF12A* siRNA treatment groups were performed using an unpaired two-tailed Student’s *t*-test. * *p* < 0.05, ** *p* < 0.01, and **** *p* < 0.0001.

We next examined whether the TWEAK/Fn14/NF-κB pathway similarly contributes to *S. salivarius*-induced fibrotic responses in LX2 cells. We first verified the suitability of the co-culture conditions for the subsequent experiments. After a 4 h exposure to logarithmic-phase *S. salivarius* at an MOI of 50:1, LX2 cell viability remained above 80% of the corresponding control level, as determined by the CCK-8 assay (Supplementary Figure S7). As observed in HepG2 cells, total NF-κB and IκBα protein levels remained unchanged among groups, whereas the *S. salivarius* group showed marked increases in p-p65, p-IκBα, and Fn14 (Figure 8A). RT-qPCR confirmed that *TNFRSF12A* and NFKB1 mRNA levels were significantly elevated in the *S. salivarius* group relative to the Model group, and these elevations were effectively reversed by BAY 11–7082 or Fn14 siRNA (Figure 8B, *p* < 0.05). To evaluate functional consequences, we measured the expression of fibrosis-related genes following pathway blockade. RT-qPCR revealed that *S. salivarius* significantly upregulated *IL6* and *TGFB* mRNA while downregulating matrix metalloproteinase-3 (*MMP3*); notably, these alterations were significantly reversed upon Fn14 siRNA transfection or NF-κB inhibition (Figure 8C,D, *p* < 0.05). To further assess hepatic stellate cell activation, we examined the protein expression of α-smooth muscle actin (α-SMA). Western blot analysis showed that α-SMA expression was increased in LX2 cells exposed to ethanol and *S. salivarius*. Importantly, this increase was markedly attenuated by either *TNFRSF12A* siRNA or treatment with the NF-κB inhibitor BAY 11–7082 (Figure 8E). Together, these results demonstrate that *S. salivarius* drives fibrotic responses in LX2 cells via Fn14/NF-κB signaling, as pathway interruption partially attenuates these pro-fibrotic effects.

## 4. Discussion

Although accumulating evidence has linked gut dysbiosis to the pathogenesis of ALD [12,13,14], the potential functions of specific gut microbes in ALD development remain poorly understood. In the present study, we found that, under ethanol exposure, *S. salivarius* impaired intestinal barrier integrity and translocated to the liver as viable bacteria. This process was associated with aggravated hepatic steatosis, inflammation, fibrosis, and metabolic disturbances. Mechanistically, *S. salivarius* promoted ALD progression through Fn14-dependent NF-κB signaling. In vitro inhibition of Fn14 or NF-κB partially attenuated *S. salivarius*-associated inflammatory and fibrogenic responses. Collectively, these findings identify *S. salivarius* as a gut-derived pathobiont that exacerbates ALD and highlight Fn14-dependent NF-κB signaling as a potential therapeutic target.

Although typically regarded as a common intestinal commensal, *S. salivarius* has been reported to be significantly enriched in various clinical cohorts [15,16,17,18], with its abundance closely correlated with hepatocellular carcinoma [15], colon cancer [16], age-related macular degeneration [17], and myocardial infarction [18]. In contrast, *S. salivarius* was reduced in severe pneumonia, and its administration ameliorated Pseudomonas aeruginosa-induced pneumonia in mice [19]. This observation suggests that the effects of *S. salivarius* may differ across diseases and contexts. In the present study, both our in vivo and in vitro experiments demonstrated that *S. salivarius* accelerates ALD progression. Notably, rather than simply promoting lipid accumulation, *S. salivarius* drives comprehensive lipid metabolic disorders. Furthermore, *S. salivarius* exhibits profound pro-inflammatory and pro-fibrotic activities. To date, only a limited number of studies have established a direct causal link between specific gut bacterial strains and the pathogenesis of ALD. Duan et al. [20] demonstrated that cytolysin-producing Enterococcus faecalis directly accelerates the onset and progression of alcoholic hepatitis by secreting cytolysin and activating downstream inflammatory pathways. Similarly, high-alcohol-producing Klebsiella pneumoniae strains in the gut can generate substantial amounts of endogenous alcohol even in the absence of exogenous alcohol intake, thereby inducing fatty liver and hepatic injury, and are considered pivotal pathogenic strains driving the progression of alcohol- and metabolism-associated liver disease [21]. Consequently, gut dysbiosis may play a fundamental role in the initiation and development of ALD by enriching opportunistic pathogens, such as *S. salivarius*.

The intestinal barrier serves as a pivotal gateway within the gut–liver axis. Under physiological conditions, the intestinal barrier limits the translocation of microbial products into the portal circulation. Barrier disruption permits their delivery to the liver, where they may exacerbate hepatic inflammation and chronic liver disease progression [22]. In our study, *S. salivarius* aggravated colonic barrier impairment, as reflected by mucus layer thinning and reduced junction-associated proteins, and this was accompanied by its detection in liver tissue by complementary approaches. Notably, intrahepatic bacterial translocation or infection has been documented in various liver diseases, including pyogenic liver abscess [23], hepatocellular carcinoma [24], and liver cirrhosis [25]; however, the precise relationship between intrahepatic bacteria and ALD remains to be fully elucidated. Our findings suggest that intestinal enrichment and possible translocation of *S. salivarius* may contribute to its pro-inflammatory and pro-fibrotic activities in the liver.

Our metabolomics and gene expression analyses together demonstrate that *S. salivarius* reshapes the hepatic metabolic landscape during ALD progression through systemic perturbations in bile acid, choline, glycerophospholipid, and lipid synthetic pathways [26,27,28]. While serum TG and TC levels were significantly elevated, key lipogenic regulators including *Srebp1c*, *Fasn*, and *Scd1* were unexpectedly downregulated alongside Acc1 upregulation [29,30,31]. Rather than reflecting typical de novo lipogenesis, this transcriptomic divergence likely represents compensatory feedback or disease stage-dependent regulatory shifts in response to severe inflammation and early fibrosis [32]. Overall, these findings indicate that *S. salivarius* exacerbates ALD not through isolated metabolic pathways, but by driving systemic lipid and metabolite disequilibrium that amplifies hepatic injury, highlighting the need for future targeted lipidomic and functional validations.

*S. salivarius* aggravated hepatic steatosis, inflammation, and fibrosis, accompanied by enhanced Tgfb1 expression and increased levels of *Il11*, *Il17*, *Il18*, and *Cxcl2*, indicating a pro-inflammatory and pro-fibrotic hepatic microenvironment. Previous studies have established that IL-17 promotes neutrophil recruitment and hepatic stellate cell (HSC) responsiveness to TGF-β [33,34,35,36,37,38], IL-18 amplifies inflammasome-mediated inflammation [39], and IL-11 acts as a master driver of organ fibrosis [40]. The concurrent elevation of Cxcl2 further implies neutrophil recruitment [41], potentially creating a cellular milieu for IL-17 production and reactive oxygen species release. Collectively, these data delineate a pathogenic cascade wherein *S. salivarius* translocation and innate immune activation recruit myeloid cells, which in turn drive HSC activation via TGF-β/IL-11/IL-17 signaling, cooperatively amplifying alcohol-induced hepatic inflammation and fibrosis [33,34,35,36,37,38].

Transcriptomic and protein analyses indicated that *S. salivarius* aggravated ALD in association with hepatic Fn14 upregulation and NF-κB activation. Although TLR2 and TLR4 protein abundance was unchanged, these findings do not exclude their potential involvement in upstream signaling. The TWEAK/Fn14 pathway has been implicated in liver injury and tissue remodeling, where Fn14 can recruit TRAF adaptor proteins and activate NF-κB-dependent inflammatory and fibrogenic responses [42,43,44]. In the present study, *Tnfsf12* and *Tnfrsf12a* mRNA levels were elevated, whereas only Fn14 protein expression was increased. This pattern is consistent with Fn14 as an injury-induced receptor that may enhance cellular responsiveness to local TWEAK [42,45]. Accordingly, *S. salivarius*-associated Fn14 upregulation may contribute to NF-κB activation, in line with the reported involvement of TWEAK/Fn14 signaling in liver fibrosis, NASH, and hepatocellular carcinoma [46,47,48]. Consistent with the reported involvement of Fn14 in hepatocyte injury and HSC activation [44,49], in vitro Fn14 silencing or NF-κB inhibition partially attenuated *S. salivarius*-associated inflammatory responses in HepG2 cells and fibrogenic responses in LX2 cells. Collectively, these findings support a role for Fn14-dependent NF-κB signaling in the inflammatory and fibrogenic effects of *S. salivarius* during the progression of ALD.

Several limitations of this study warrant acknowledgement. First, while we validated the pathogenic role of *S. salivarius* primarily through oral gavage-mediated supplementation, we did not employ antibiotic-mediated depletion or germ-free animal colonization models to evaluate the necessity of endogenous *S. salivarius* in the pathogenesis of ALD. Second, the specific bacterial effector molecules responsible for driving intestinal barrier disruption remain to be identified; future investigations incorporating mutant strain screening or targeted metabolite identification are required to elucidate these active components. Third, only female C57BL/6 mice were included in this study. Given that sex-dependent differences may influence susceptibility to alcohol-induced hepatic injury and phenotype severity, the extent to which these findings apply to male mice remains to be established. Future studies should replicate these experiments in male mice and evaluate sex as an important biological variable. Fourth, this study did not include an *S. salivarius*-alone group without ethanol exposure. Therefore, we could not determine whether *S. salivarius* independently induces liver injury or interacts with ethanol exposure to worsen ALD-related pathology. Future studies including an *S. salivarius*-alone control group are warranted to clarify its effects in the absence of ethanol. Finally, the effects of the *S. salivarius* culture supernatant were assessed only in HepG2-CYP2E1 cells and not in LX2 cells; therefore, its potential effects on hepatic stellate-cell activation and fibrotic responses remain undetermined.

## 5. Conclusions

In conclusion, our findings demonstrate that *S. salivarius* disrupts intestinal barrier integrity under ethanol exposure and translocates to the liver. This process is associated with hepatic inflammation, fibrosis, and metabolic dysregulation and may involve Fn14-dependent NF-κB signaling, thereby exacerbating ALD progression. These findings suggest that *S. salivarius* and Fn14-dependent NF-κB signaling may be potential therapeutic targets for ALD.

## Data Availability

The RNA-seq data have been deposited in the NCBI Gene Expression Omnibus (GEO) under accession number GSE348727. The metabolomics data have been deposited in Metabolomics Workbench under Study ID ST005269. The data that support the findings of this study are available from the corresponding author upon reasonable request.

## Supplementary Materials

The following supporting information can be downloaded at: https://www.mdpi.com/article/10.3390/microorganisms14102297/s1, Table S1. Primer sequences used for real-time quantitative PCR in mouse tissues, cultured cells, and *S. salivariu*. Table S2. Parameters of the *S. salivarius*-specific qPCR standard curve. Figure S1. BLAST-based identification of the clinical Streptococcus salivarius isolate using the 16S rRNA gene sequence. Figure S2. Standard curve for the absolute quantification of *S. salivarius* DNA by qPCR. Figure S3. Metabolomic profiling reveals *S. salivarius*-induced metabolic alterations in mouse liver tissue, related to Figure 4. (A) Partial least squares discriminant analysis (PLS-DA) score plot. (B) K-means clustering of metabolites across experimental groups. (C) Volcano plot of differentially abundant metabolites. (D) Heatmap of hierarchical clustering for differentially abundant metabolites. (E) Classification of identified metabolites. Figure S4. Effects of *S. salivarius* on the hepatic transcriptome in mice with alcoholic liver disease, related to Figure 6. (A) Principal component analysis (PCA); (B) Heatmap of hierarchical clustering analysis of differentially expressed genes; (C) Bubble plot of functional enrichment analysis. Figure S5. Effects of *S. salivarius* culture supernatant on hepatocellular injury and inflammatory gene expression in HepG2 cells. Figure S6. Gentamicin protection assay to assess the association of viable *S. salivarius* with HepG2-CYP2E1 cells. Figure S7. Effects of *S. salivarius* co-culture on the viability of HepG2-CYP2E1 and LX2 cells.
