# Supplementary material for: Gut-to-Liver Escape of Viable Streptococcus salivarius Fuels Alcoholic Liver Disease via Fn14-Dependent NF-κB Signaling
Source: Microorganisms. 2026 Oct 9;14(10):2297. doi: 10.3390/microorganisms14102297 (PMC13707417; doi:10.3390/microorganisms14102297)

Fig. 2: Raw image of molecular weight marker
Membrane: PVDF
Group: [C57BL/6 mouse liver tissue]
Control, Model, *S. salivarius*

MW Marker: 3-Color Prestained Protein Ladder, 13–250 kDa, SparkJade, Cat. No. EC1020

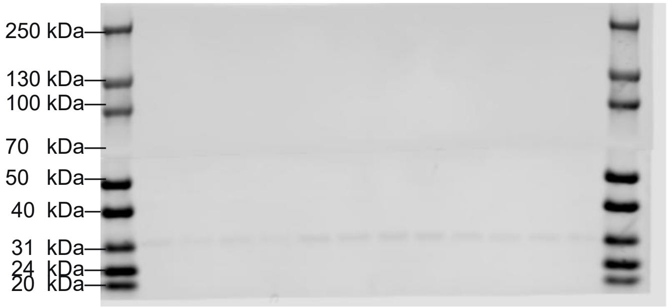

Beta Actin
Proteintech 20536-1-AP
42 kDa

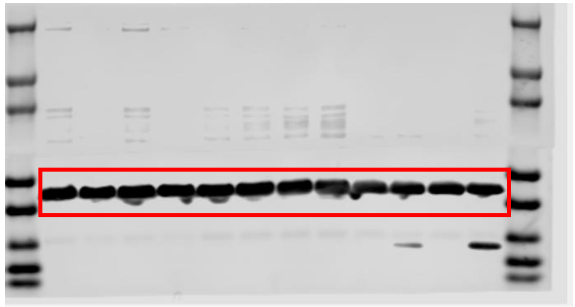

ZO-1
Proteintech 21773-1-AP
230 kDa

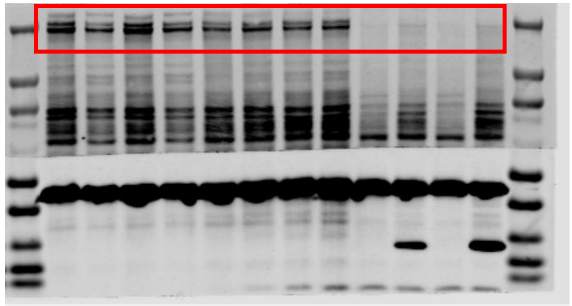

MW Marker: PageRuler™ Prestained Protein Ladder, 10 to 180 kDa, Thermo Scientific™, Cat. No. 26616

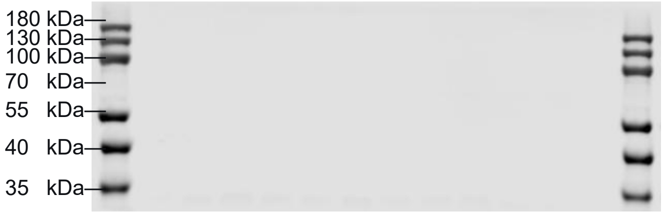

Beta Actin
Proteintech 20536-1-AP
42 kDa

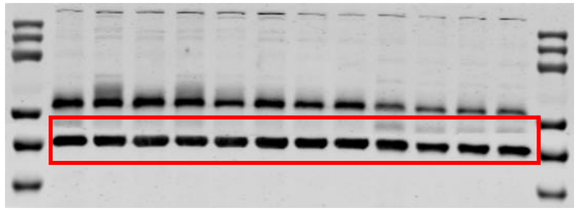

Occludin
Proteintech 27260-1-AP
59 kDa

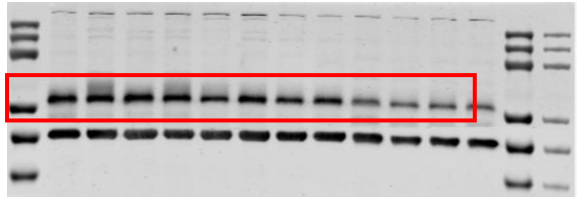

MW Marker: PageRuler™ Prestained Protein Ladder, 10 to 180 kDa, Thermo Scientific™, Cat. No. 26616

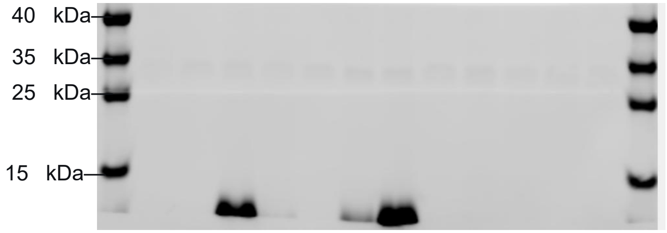

Beta Actin
Proteintech 20536-1-AP
42 kDa

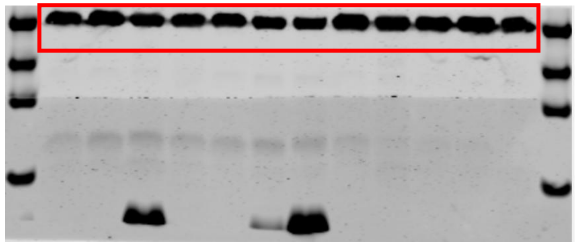

Claudin 1
Proteintech 28674-1-AP
20 kDa

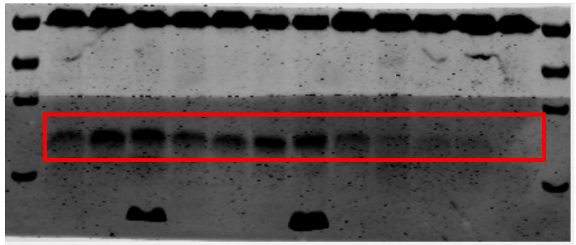

Fig. 5: Raw image of molecular weight marker
Membrane: PVDF
MW Marker: PageRuler™ Prestained Protein Ladder, 10 to 180 kDa, Thermo Scientific™, Cat. No. 26616
Group: [C57BL/6 mouse liver tissue]
Control, Model, *S. salivarius*

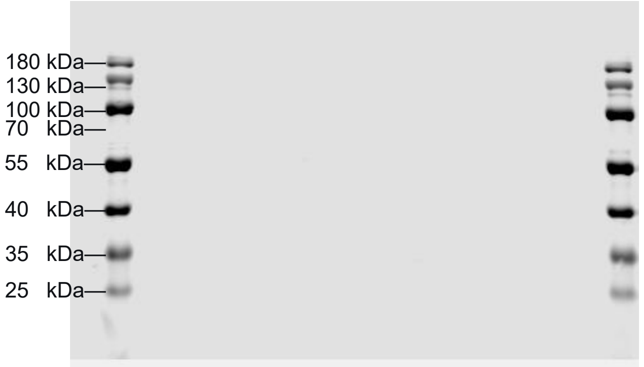

Beta Actin
Proteintech 20536-1-AP
42 kDa

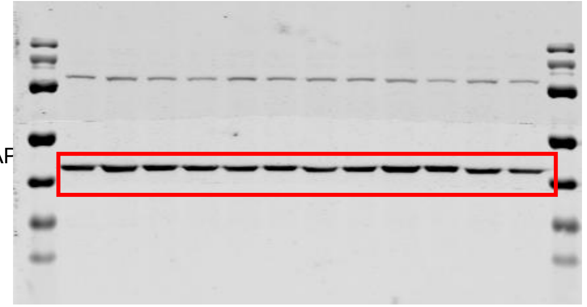

TLR4
Proteintech 19811-1-AP
110 kDa

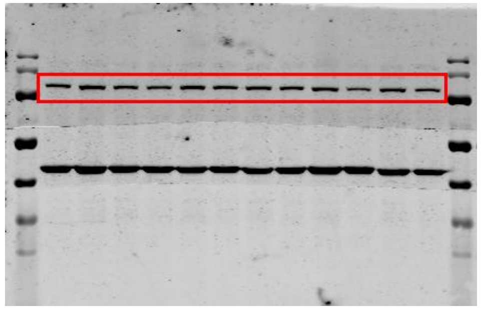

Beta Actin
Proteintech
20536-1-AP
42 kDa

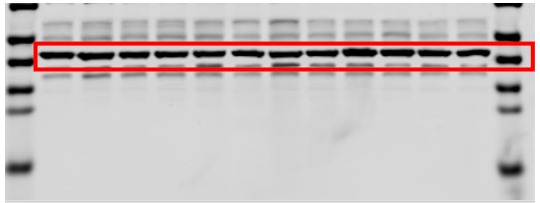

NF-κB p65
Proteintech
80979-1-RR
65 kDa

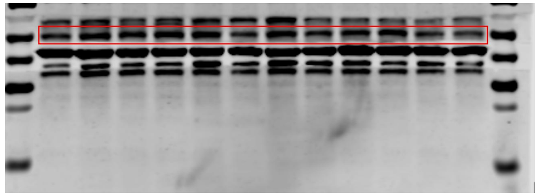

IkB Alpha
Proteintech
10268-1-AP
36 kDa

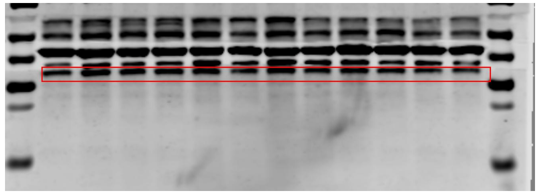

Beta Actin
Proteintech
20536-1-AP
42 kDa

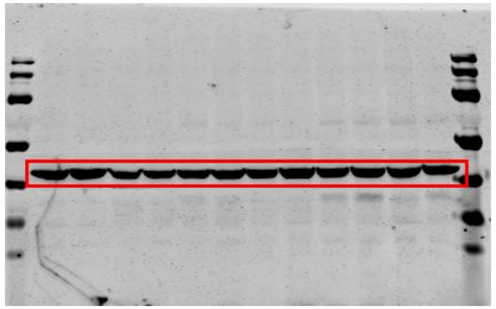

Phospho-NF-κB
p65 (Ser468)
Proteintech
82335-1-RR
65 kDa

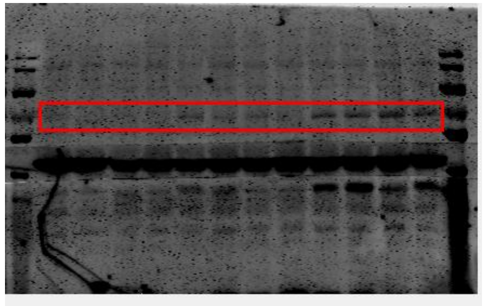

Phospho-IkB Alpha
(Ser32/36)
Proteintech 82349-1-RR
36 kDa

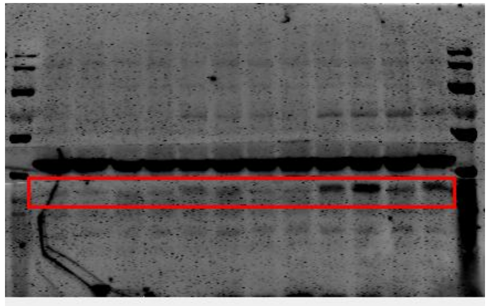

Beta Actin
Proteintech
20536-1-AP
42 kDa

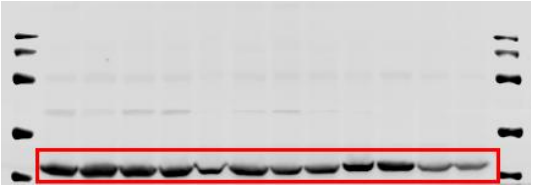

TLR2
CST #13744
100 kDa

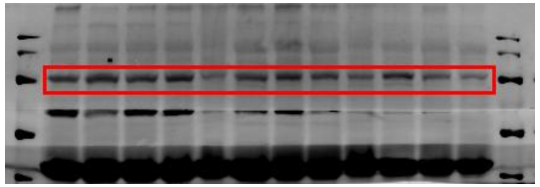

Fig. 5: Raw image of molecular weight marker
Membrane: PVDF
MW Marker: PageRuler™ Prestained Protein Ladder, 10 to 180 kDa, Thermo Scientific™, Cat. No. 26616
Group: [C57BL/6 mouse liver tissue]
Control, Model, *S. salivarius*

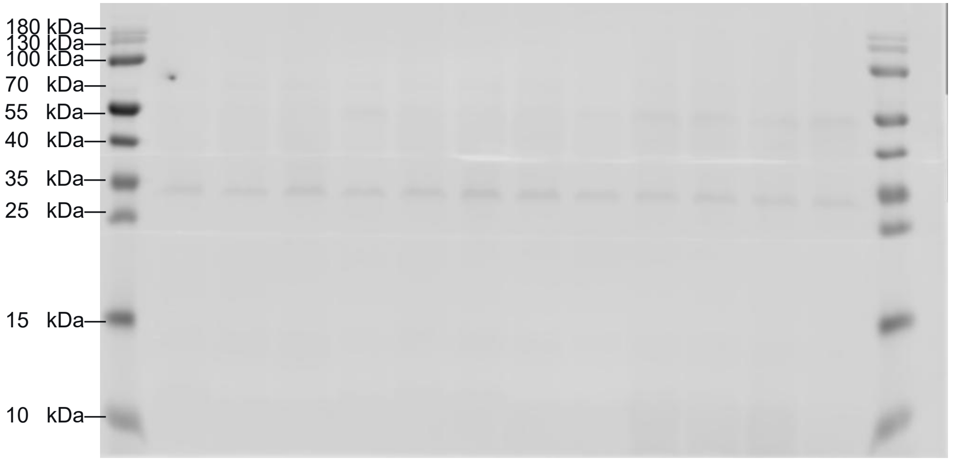

Beta Actin
Proteintech
20536-1-AP
42 kDa

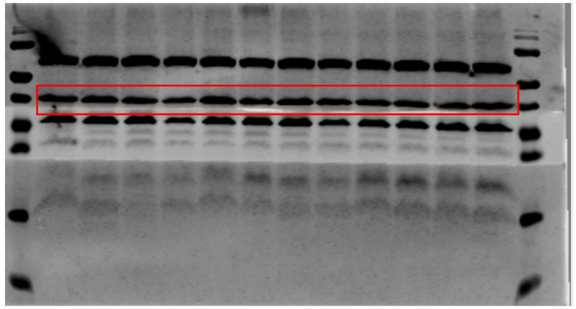

TWEAK
abcam ab37170
36 kDa

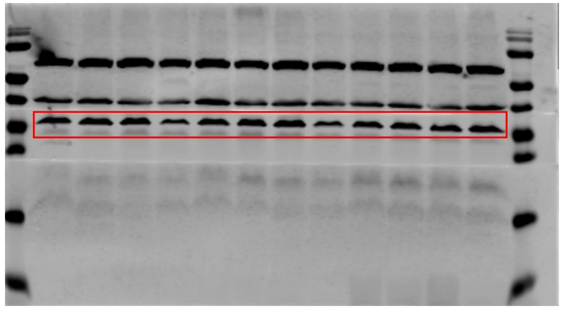

TWEAKR/FN14
abcam ab109365
17 kDa

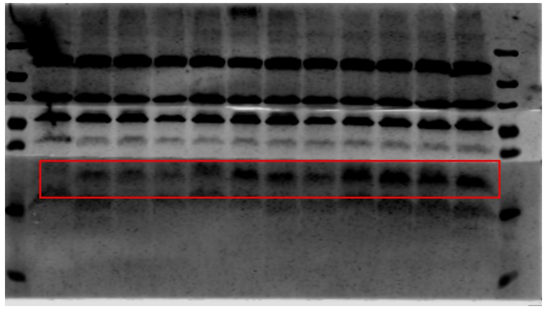

Fig. 6: Raw image of molecular weight marker
Membrane: PVDF
MW Marker: PageRuler™ Prestained Protein Ladder, 10 to 180 kDa, Thermo Scientific™, Cat. No. 26616
Group: [HepG2-CYP2E1]
Control, Model, *S. salivarius*, BAY 11-7082, Fn14 KO

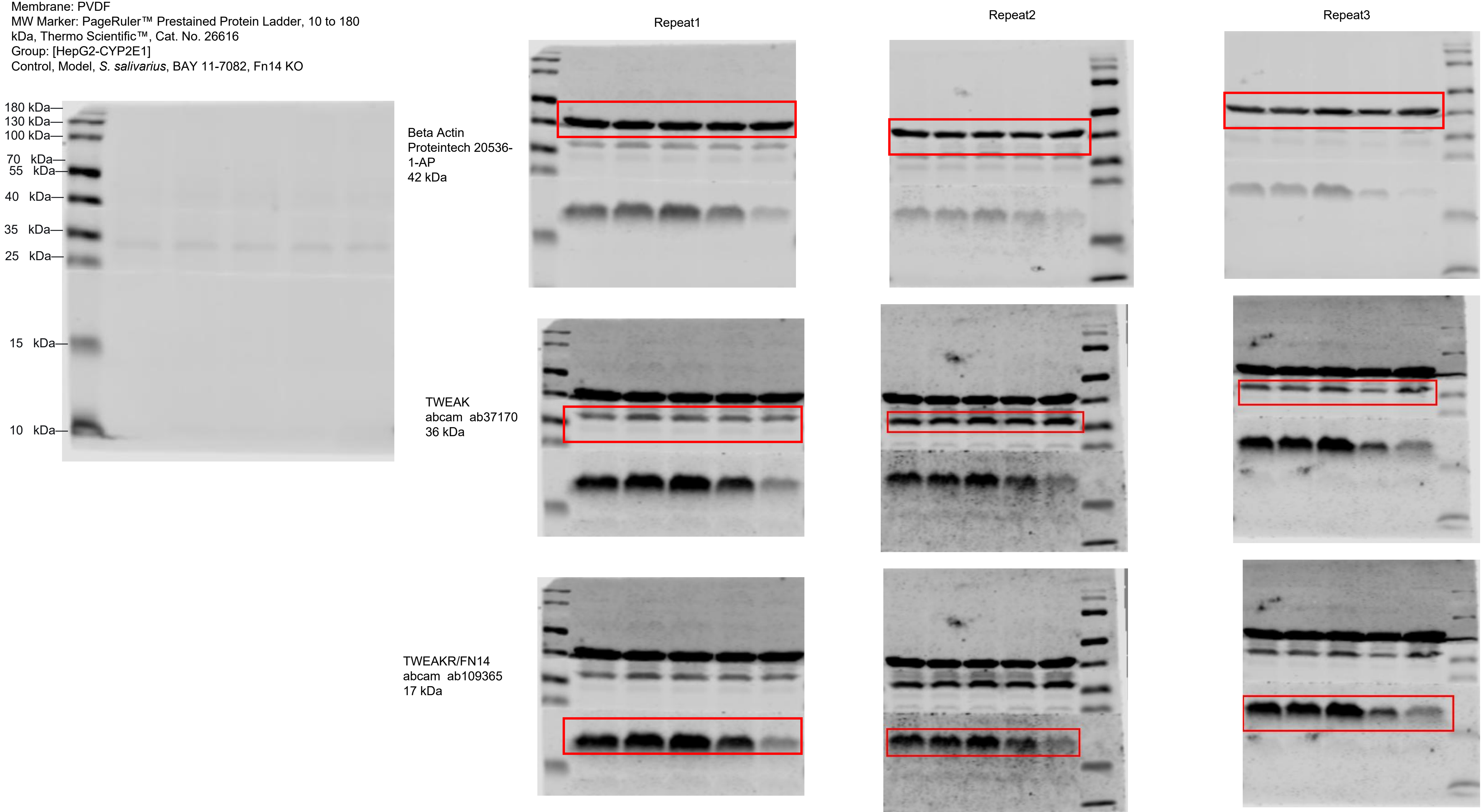

Fig. 6: Raw image of molecular weight marker
Membrane: PVDF
MW Marker: PageRuler™ Prestained Protein Ladder, 10 to 180 kDa, Thermo Scientific™, Cat. No. 26616
Group: [HepG2-CYP2E1]
Control, Model, *S. salivarius*, BAY 11-7082, Fn14 KO

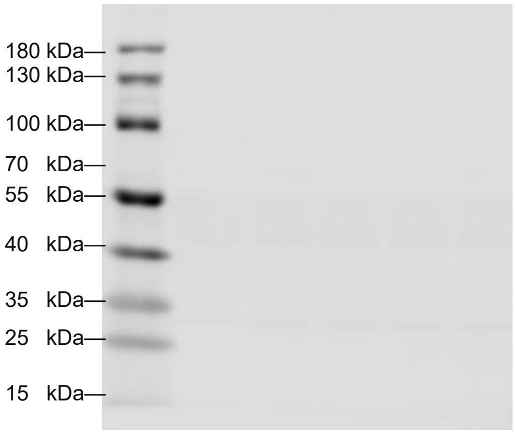

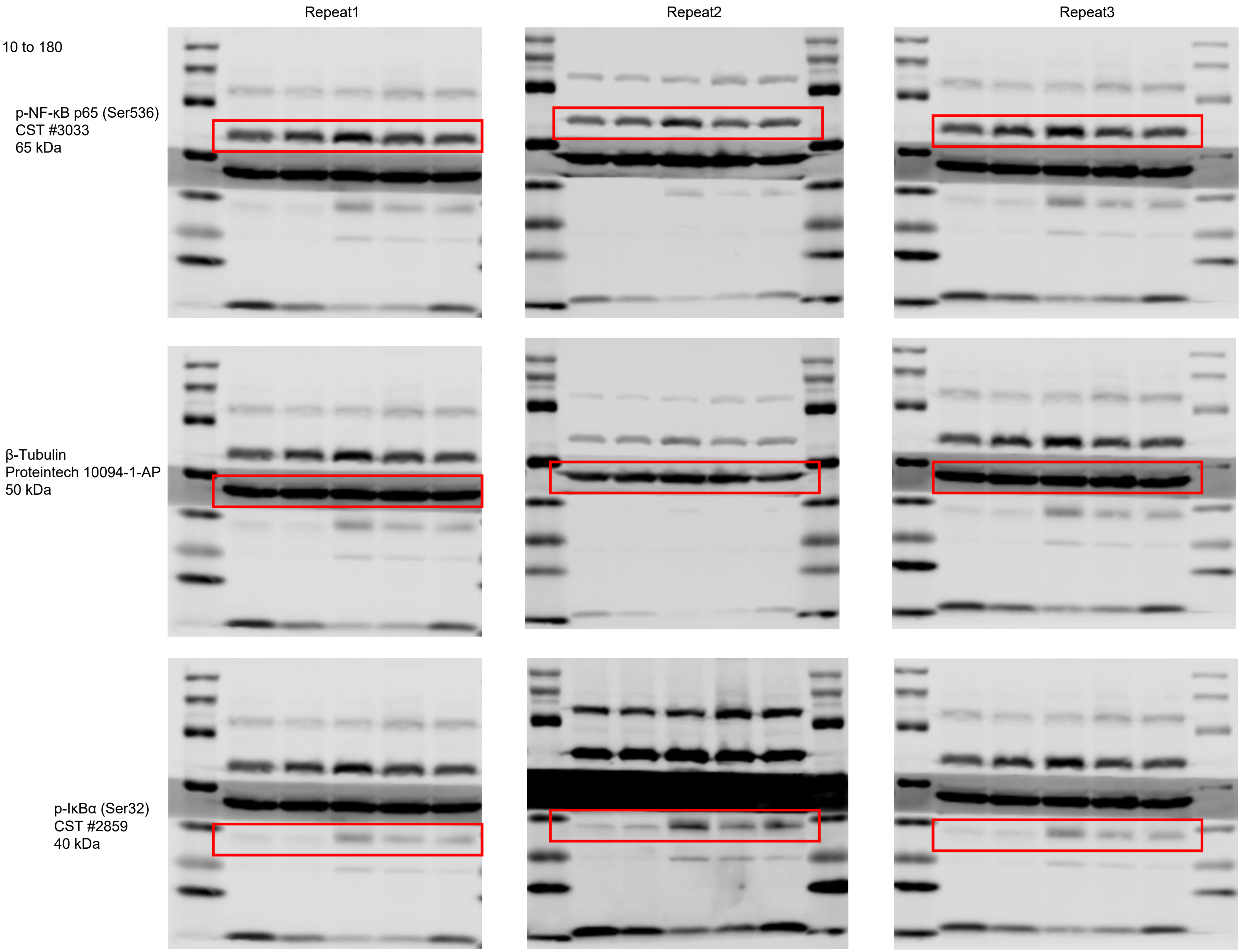

Fig. 6: Raw image of molecular weight marker
Membrane: PVDF
MW Marker: PageRuler™ Prestained Protein Ladder, 10 to 180 kDa, Thermo Scientific™, Cat. No. 26616
Group: [HepG2-CYP2E1]
Control, Model, *S. salivarius*, BAY 11-7082, Fn14 KO

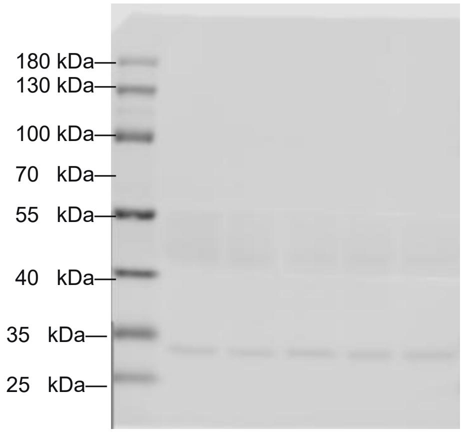

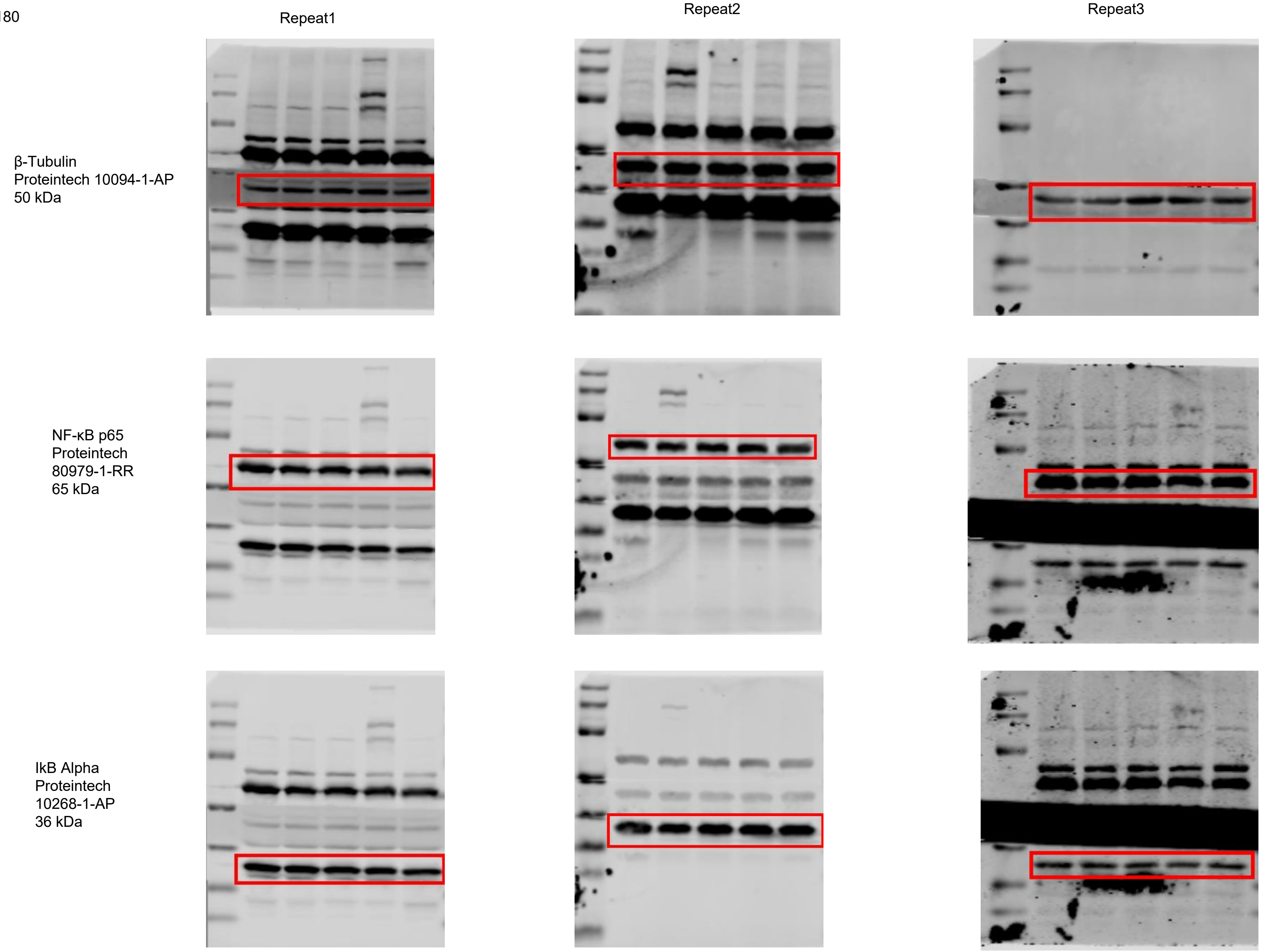

Fig. 7: Raw image of molecular weight marker
Membrane: PVDF
MW Marker: PageRuler™ Prestained Protein Ladder, 10 to 180 kDa, Thermo Scientific™, Cat. No. 26616
Group: [LX2]
Control, Model, *S. salivarius*, BAY 11-7082, Fn14 KO

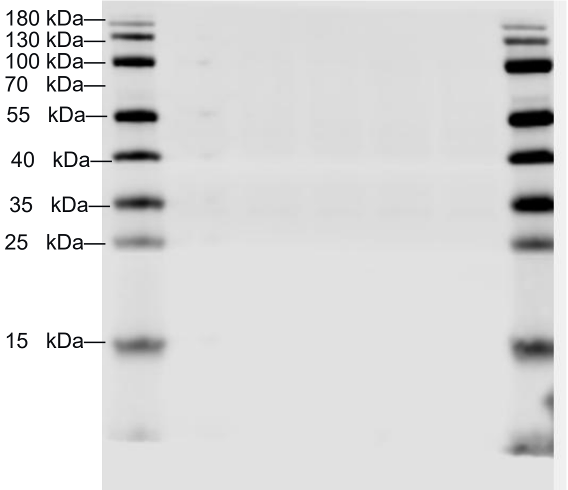

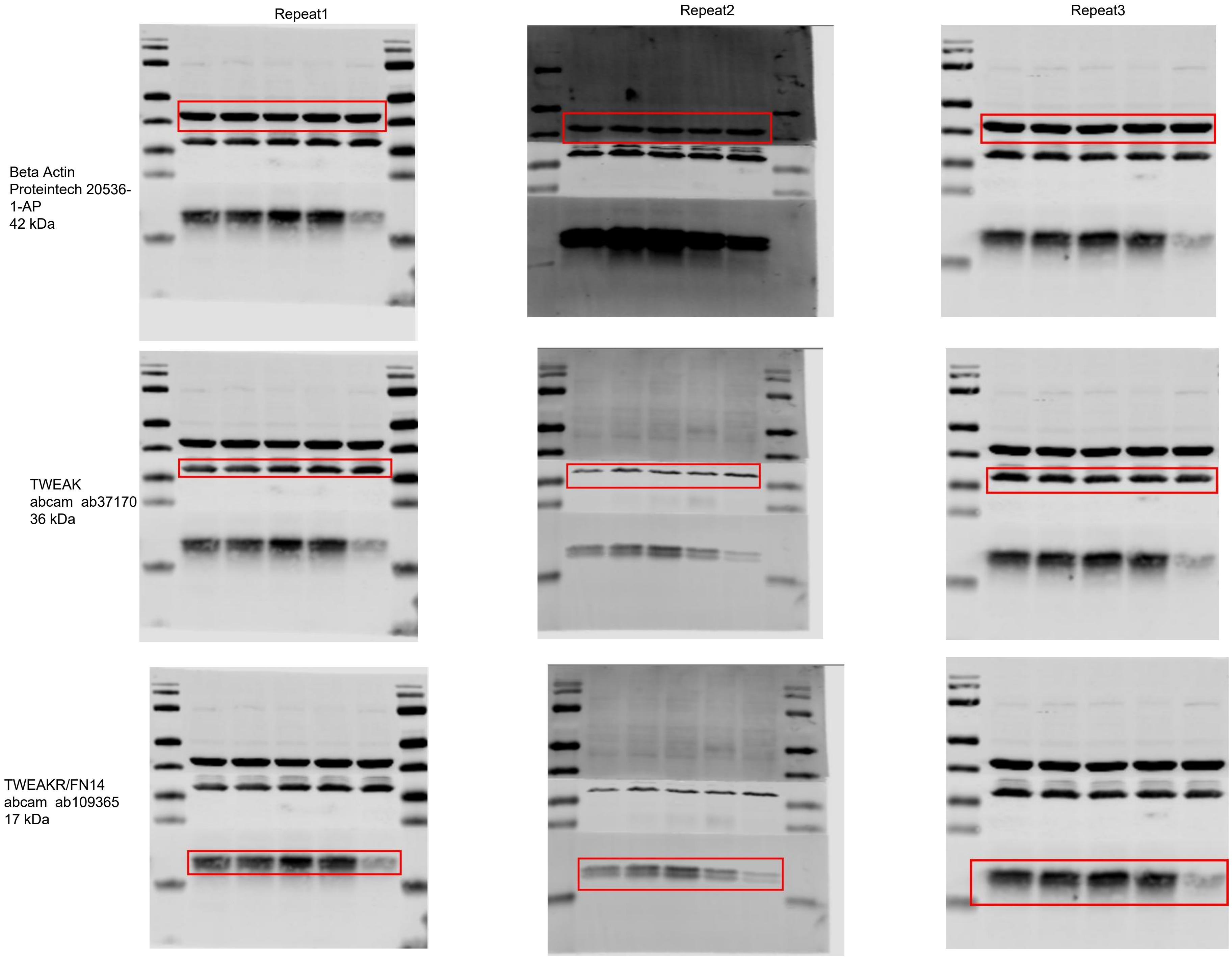

Fig. 7: Raw image of molecular weight marker
Membrane: PVDF
MW Marker: PageRuler™ Prestained Protein Ladder, 10 to 180 kDa, Thermo Scientific™, Cat. No. 26616
Group: [LX2]
Control, Model, *S. salivarius*, BAY 11-7082, Fn14 KO

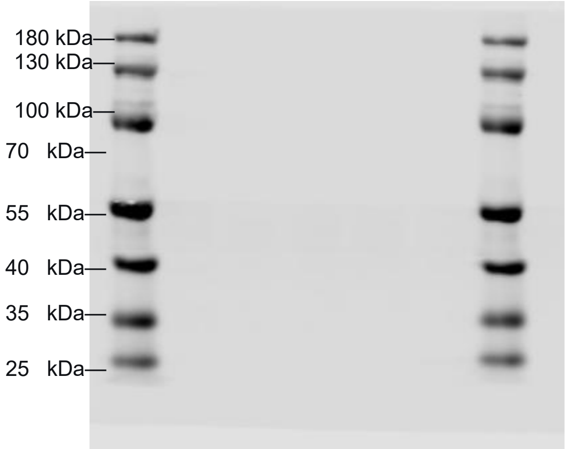

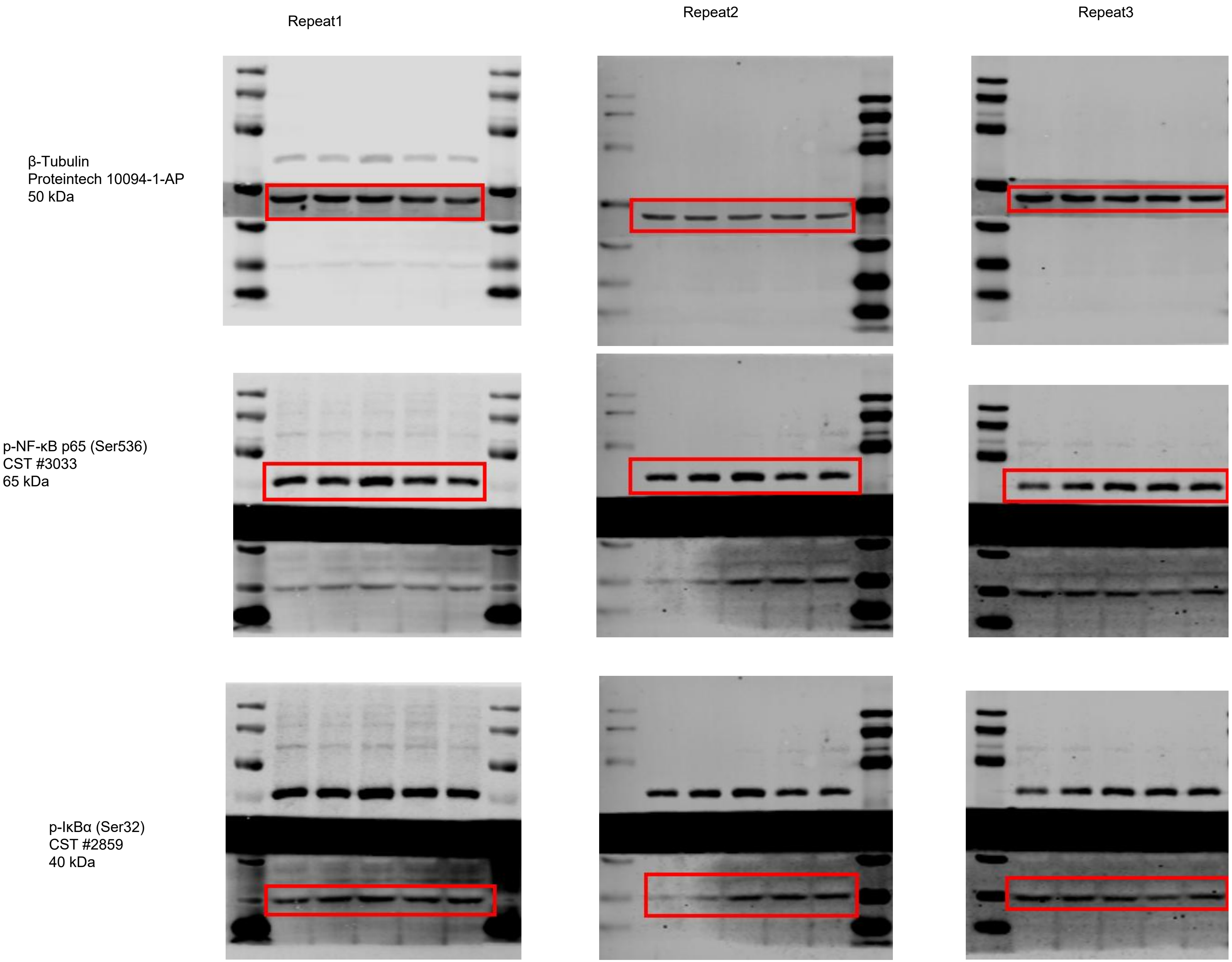

Fig. 7: Raw image of molecular weight marker
Membrane: PVDF
MW Marker: PageRuler™ Prestained Protein Ladder, 10 to 180 kDa, Thermo Scientific™, Cat. No. 26616
Group: [LX2]
Control, Model, *S. salivarius*, BAY 11-7082, Fn14 KO

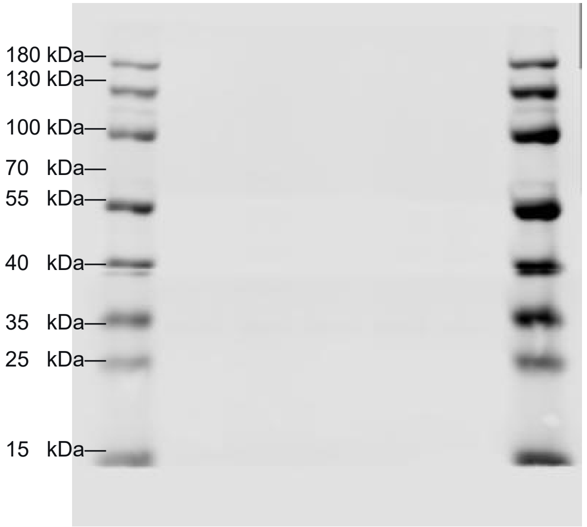

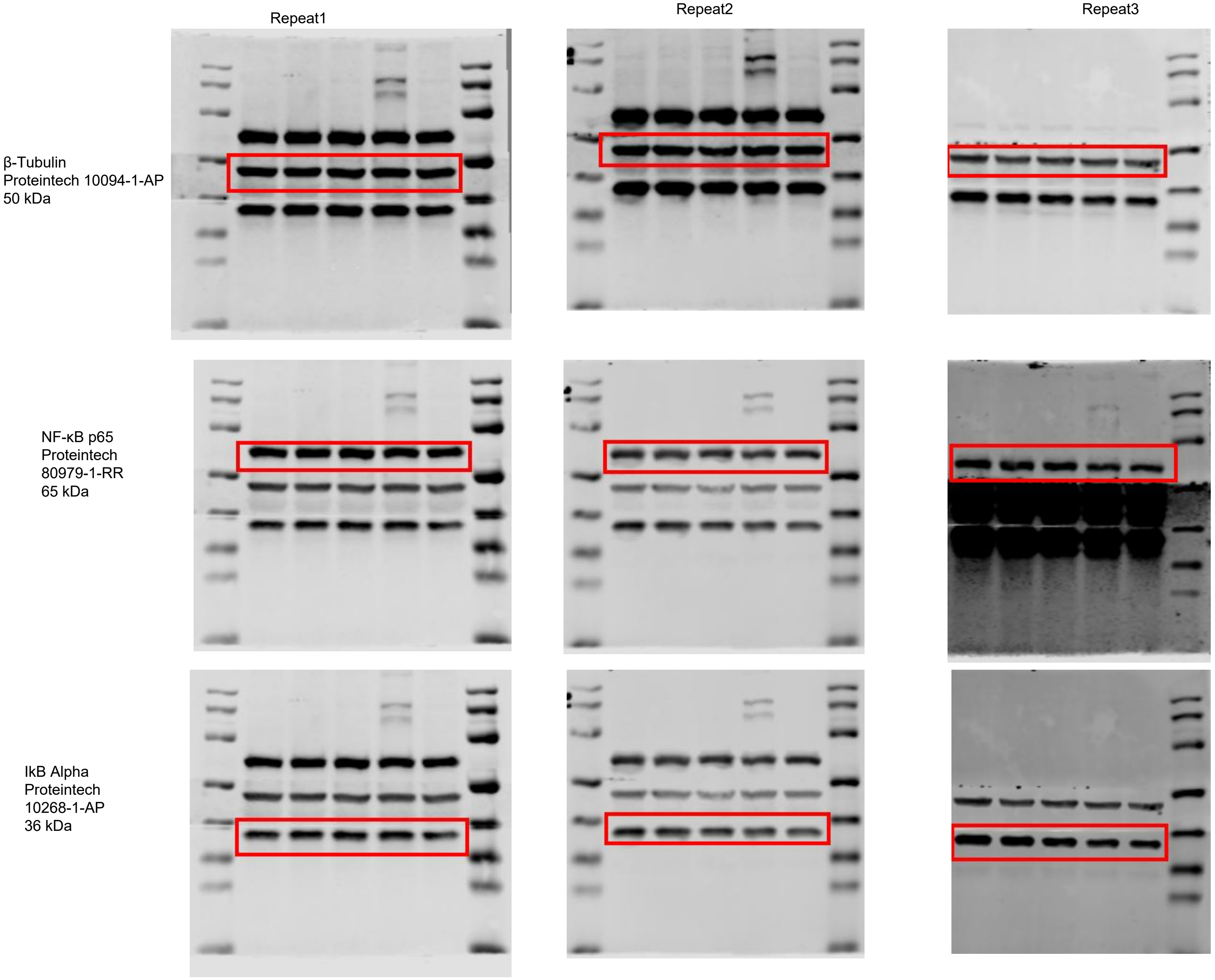

Fig. 7: Raw image of molecular weight marker
Membrane: PVDF
MW Marker: PageRuler™ Prestained Protein Ladder, 10 to 180 kDa, Thermo Scientific™, Cat. No. 26616
Group: [LX2]
Control, Model, *S. salivarius*, BAY 11-7082, Fn14 KO

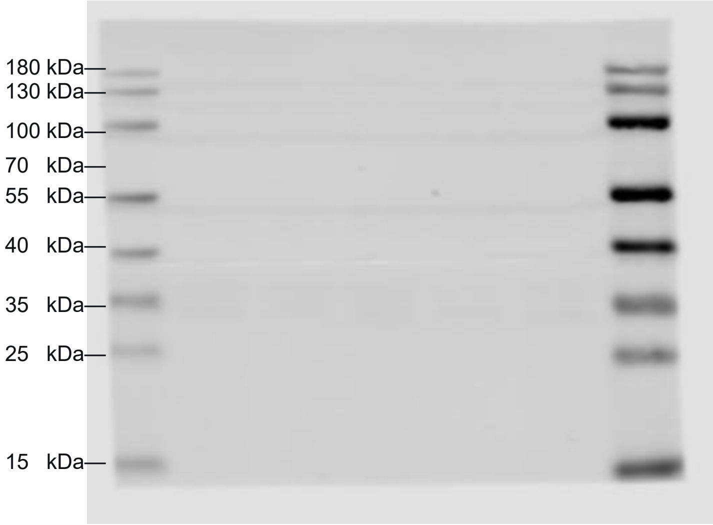

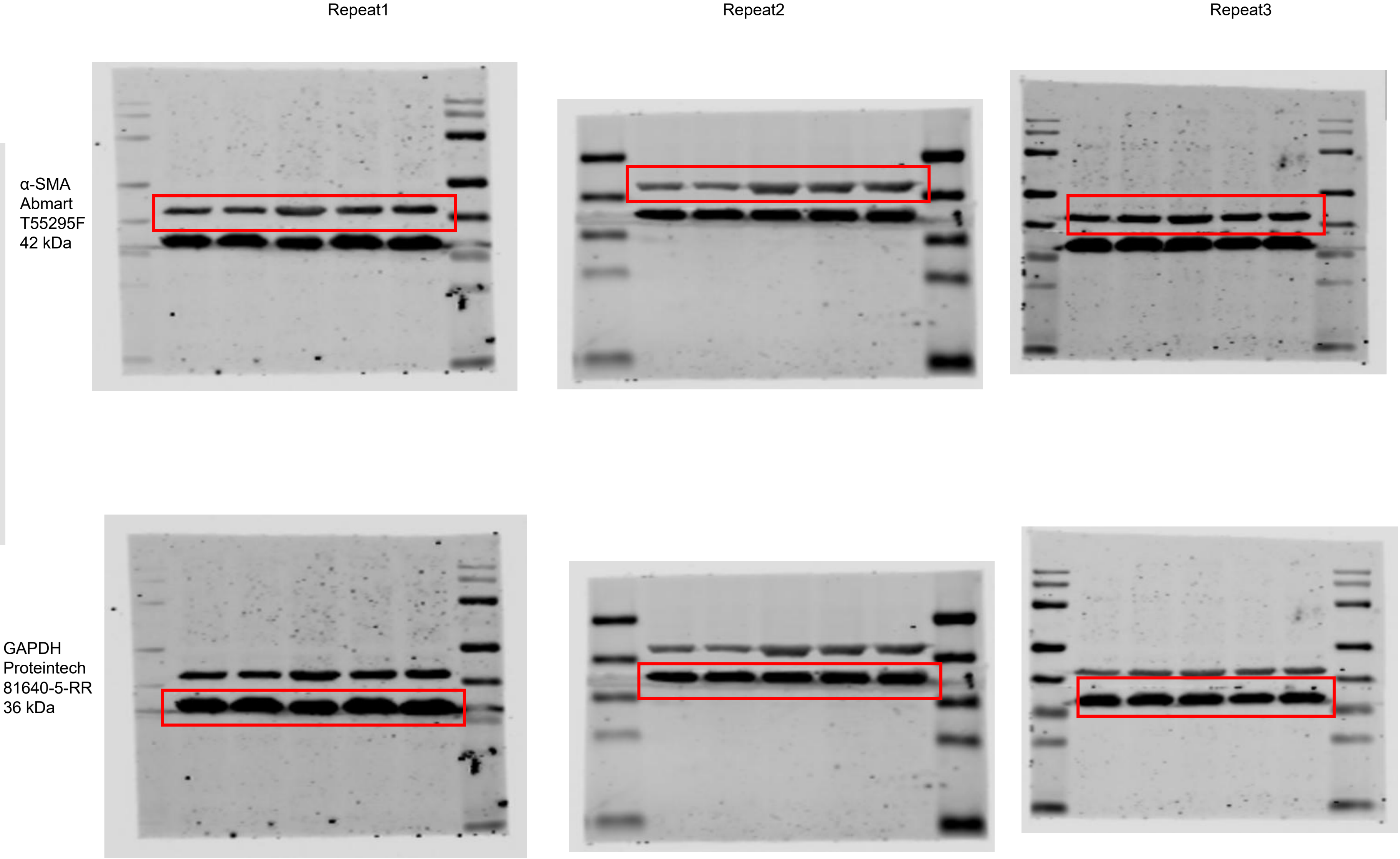

Raw Image File – Fig. 2. Alcian blue staining of mouse colon tissue.
Group: C57BL/6 mouse colon tissue. Control (n=6), Model (n=8), *S. salivarius* (n=11). Scale bar = 100 μm. Magnification: 20×.

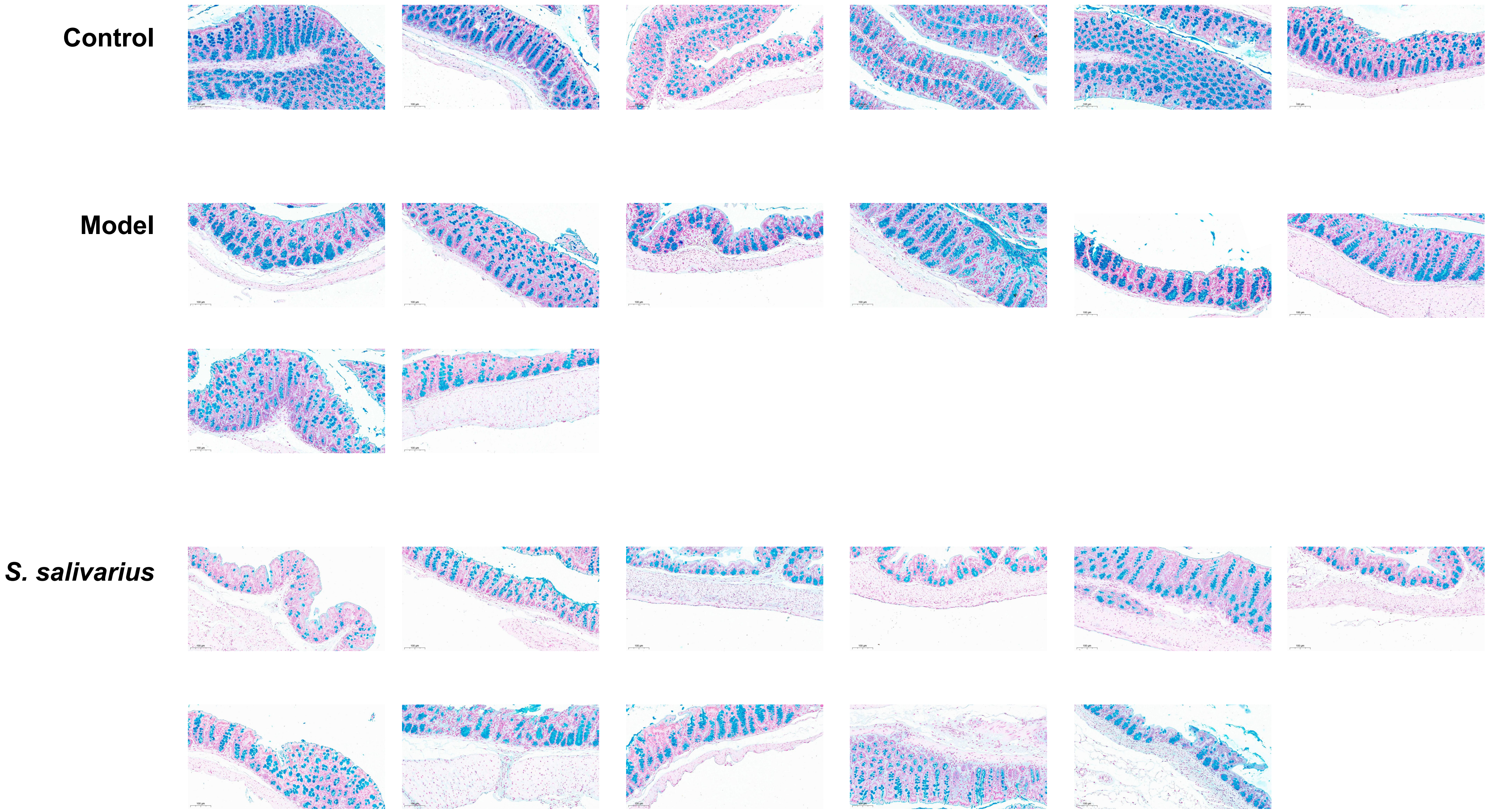

Raw Image File – Fig. 4. Oil Red O staining of mouse liver tissue.
Group: C57BL/6 mouse liver tissue. Control (n=6), Model (n=8), *S. salivarius* (n=11). Scale bar = 200 μm. Magnification: 10×.

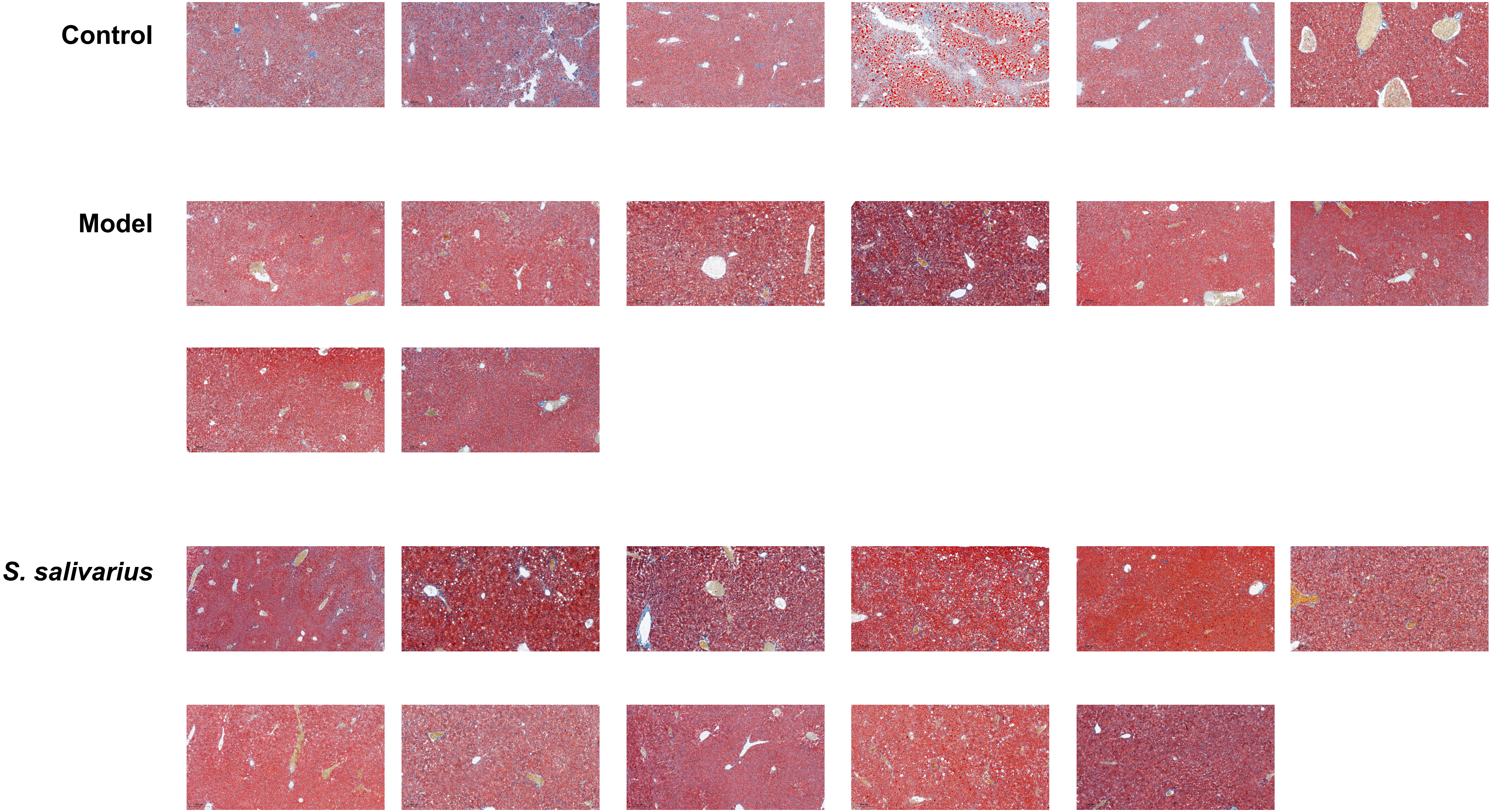

Raw Image File – Fig. 5.Sirius Red staining of mouse liver tissue.
Group: C57BL/6 mouse liver tissue. Control (n=6), Model (n=8), *S. salivarius* (n=11). Scale bar = 50 μm. Magnification: 20×.

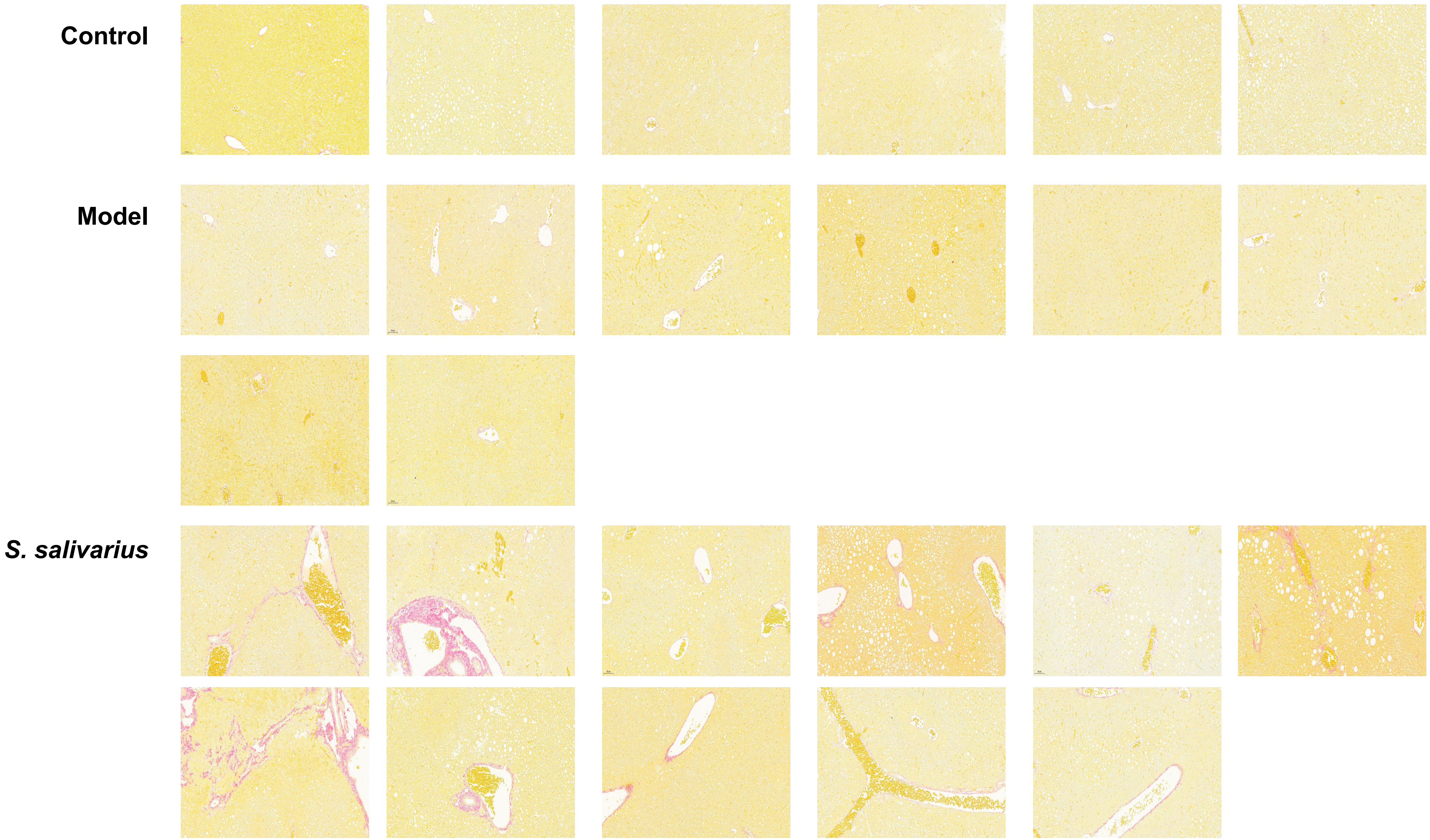

Supplement: Supplementary file 1 [file microorganisms-14-02297-s001.zip › Original Western Blot and Microscope Images-ID microorganisms-4578386.pdf]
