# Supplementary material for: Gut-to-Liver Escape of Viable Streptococcus salivarius Fuels Alcoholic Liver Disease via Fn14-Dependent NF-κB Signaling
Source: Microorganisms. 2026 Oct 9;14(10):2297. doi: 10.3390/microorganisms14102297 (PMC13707417; doi:10.3390/microorganisms14102297)
Supplement: Supplementary file 1 [file microorganisms-14-02297-s001.zip › Supplementary Figure.pdf]

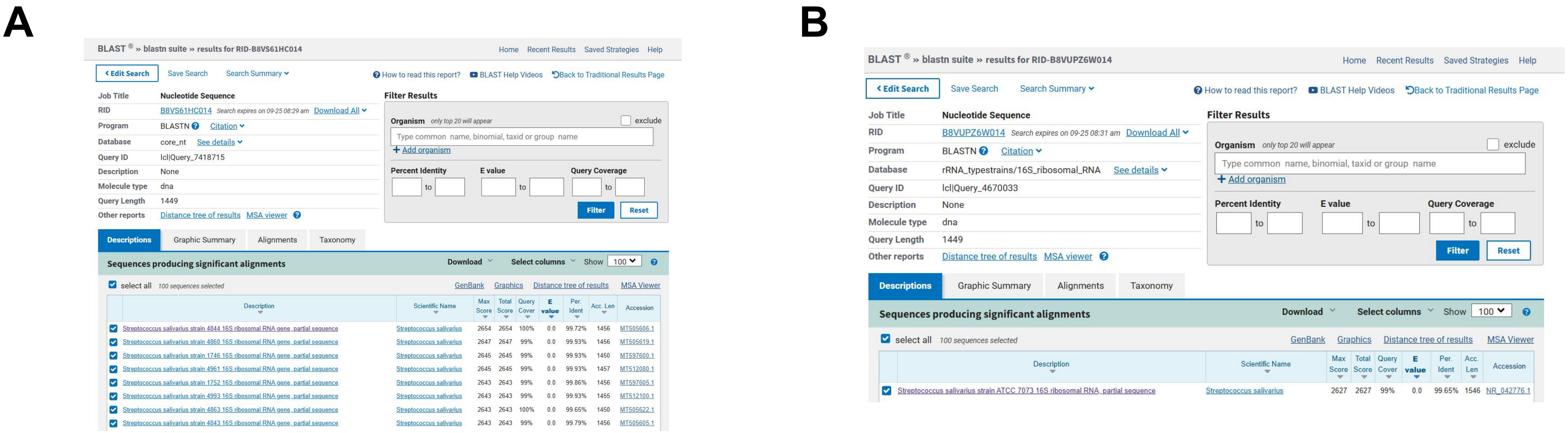

Figure S1. BLAST-based identification of the clinical *Streptococcus salivarius* isolate using the 16S rRNA gene sequence.
(A) BLAST alignment result obtained from the NCBI Core Nucleotide Database, showing that the 16S rRNA gene sequence of the clinical isolate had the closest match to Streptococcus salivarius strain 4844. (B) BLAST alignment result obtained from the NCBI 16S rRNA sequence database, showing that the closest matching reference sequence was annotated as Streptococcus salivarius strain ATCC 7073.

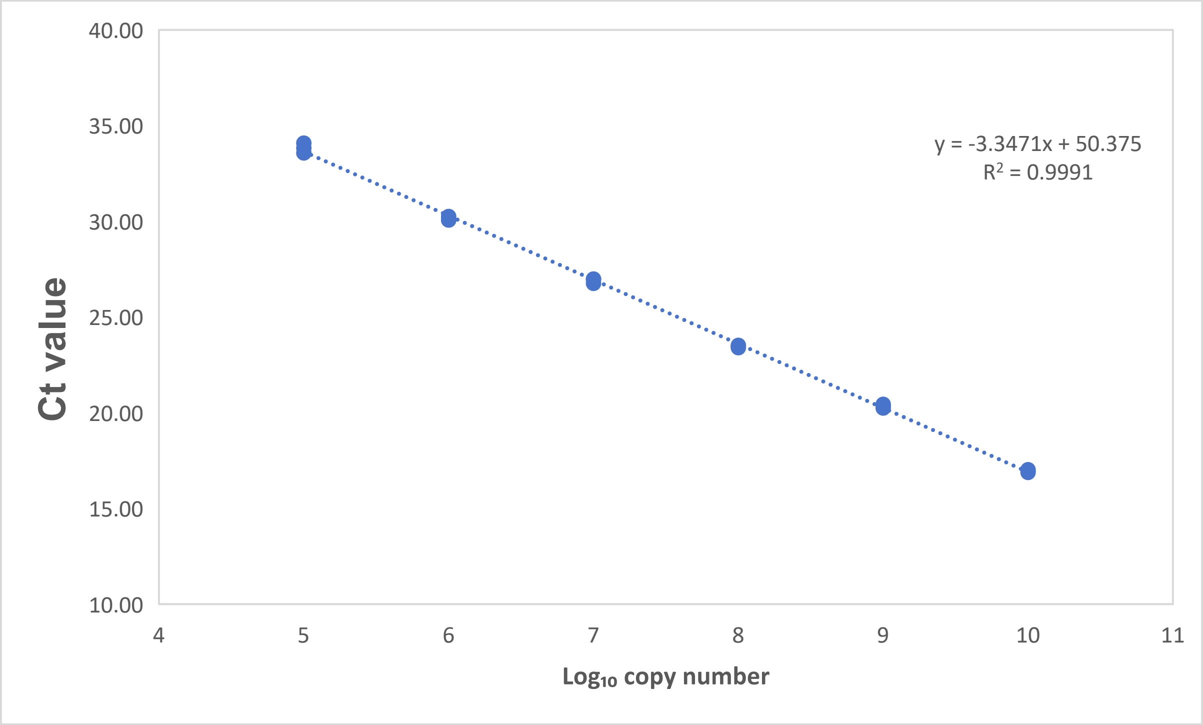

Figure S2. Standard curve for the absolute quantification of S. salivarius DNA by qPCR. The standard curve was generated using serially diluted S. salivarius genomic DNA. The Ct values were plotted against the corresponding DNA copy numbers.

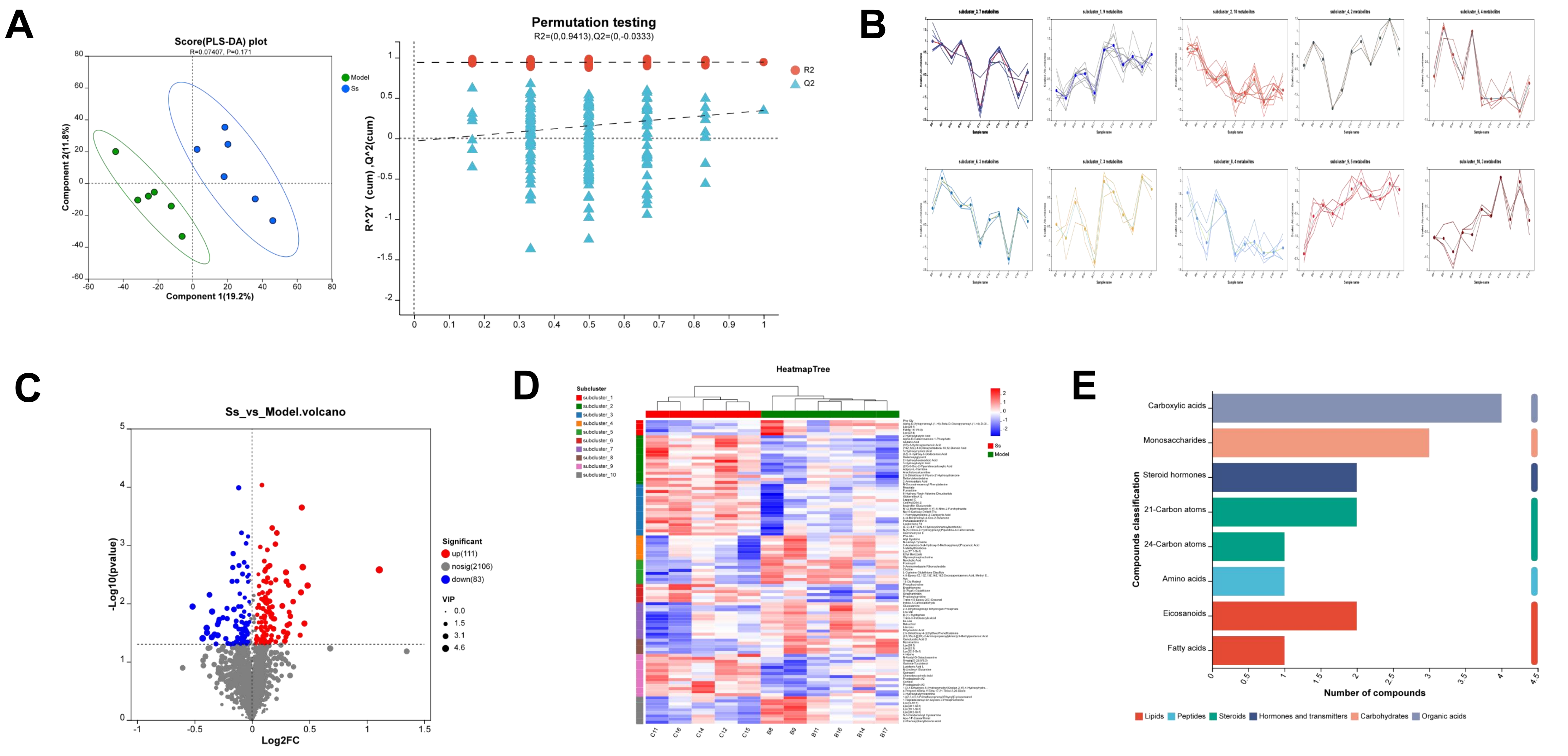

Figure S3. Metabolomic profiling reveals S. salivarius-induced metabolic alterations in mouse liver tissue, related to Figure 4. (A) Partial least squares-discriminant analysis (PLS-DA) score plot. **(B)** K-means clustering of metabolites across experimental groups. **(C)** Volcano plot of differentially abundant metabolites. **(D)** Heatmap of hierarchical clustering for differentially abundant metabolites. **(E)** Classification of identified metabolites.

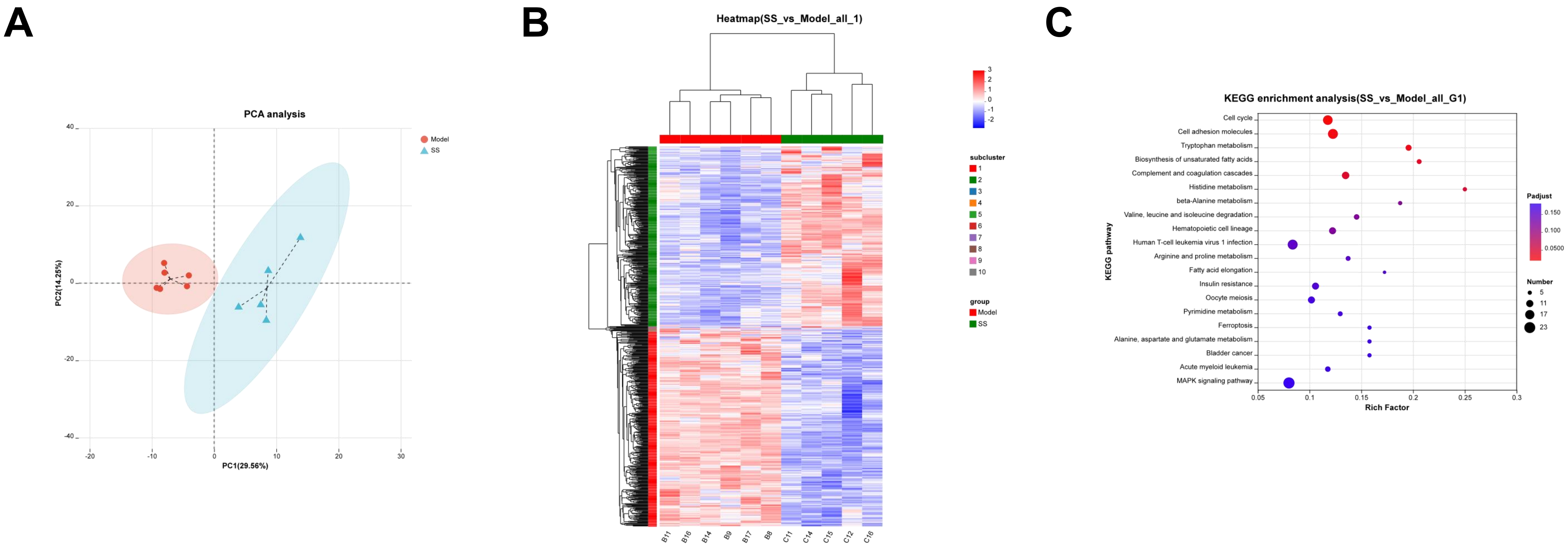

Figure S4. Effects of *S. salivarius* on the hepatic transcriptome in mice with alcoholic liver disease, related to Figure 6. (A) Principal component analysis (PCA); **(B)** Heatmap of hierarchical clustering analysis of differentially expressed genes; **(C)** Bubble plot of functional enrichment analysis.

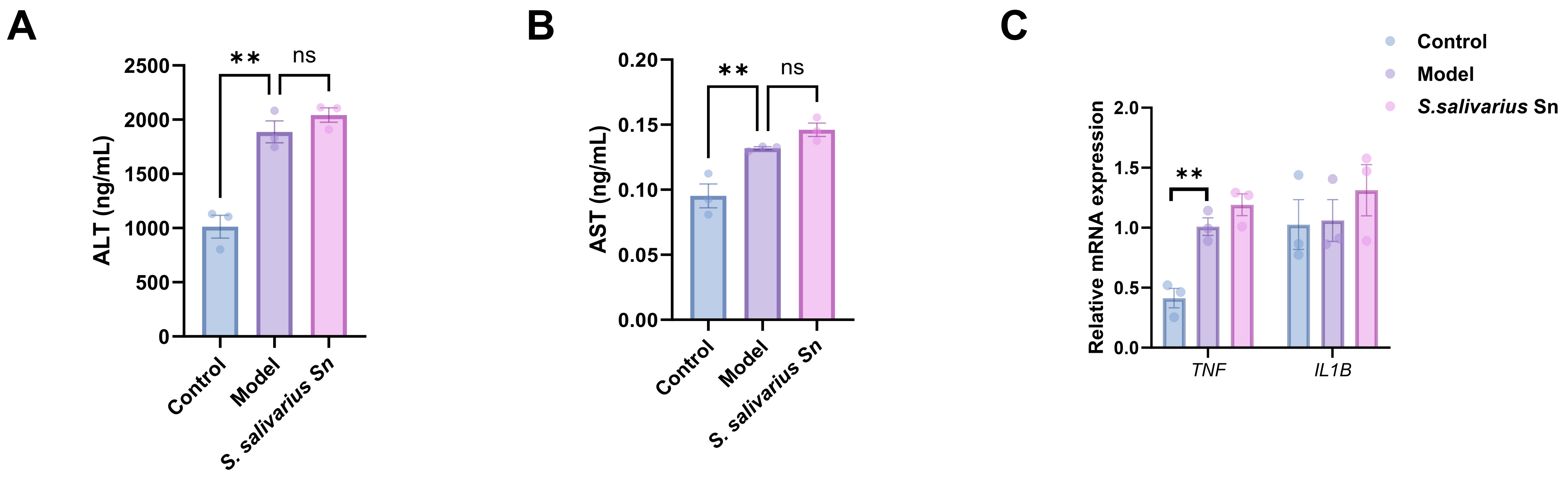

Figure S5. Effects of S. salivarius culture supernatant on hepatocellular injury and inflammatory gene expression in HepG2 cells.

(A-B) Alanine aminotransferase (ALT) and aspartate aminotransferase (AST) levels in the culture supernatants of HepG2-CYP2E1 cells. (C) Relative mRNA expression levels of *TNF* and *IL1B* in HepG2-CYP2E1 cells, as determined by quantitative reverse-transcription PCR (RT-qPCR).

Data are presented as mean ± SEM (n = 3). Statistical analysis was performed using one-way ANOVA followed by Dunnett's multiple-comparisons test for comparisons among the Control, Model, and S. salivarius groups, with the Model group as the reference group. *P < 0.05, **P < 0.01, ***P < 0.001 and ****P < 0.0001; ns, not significant.

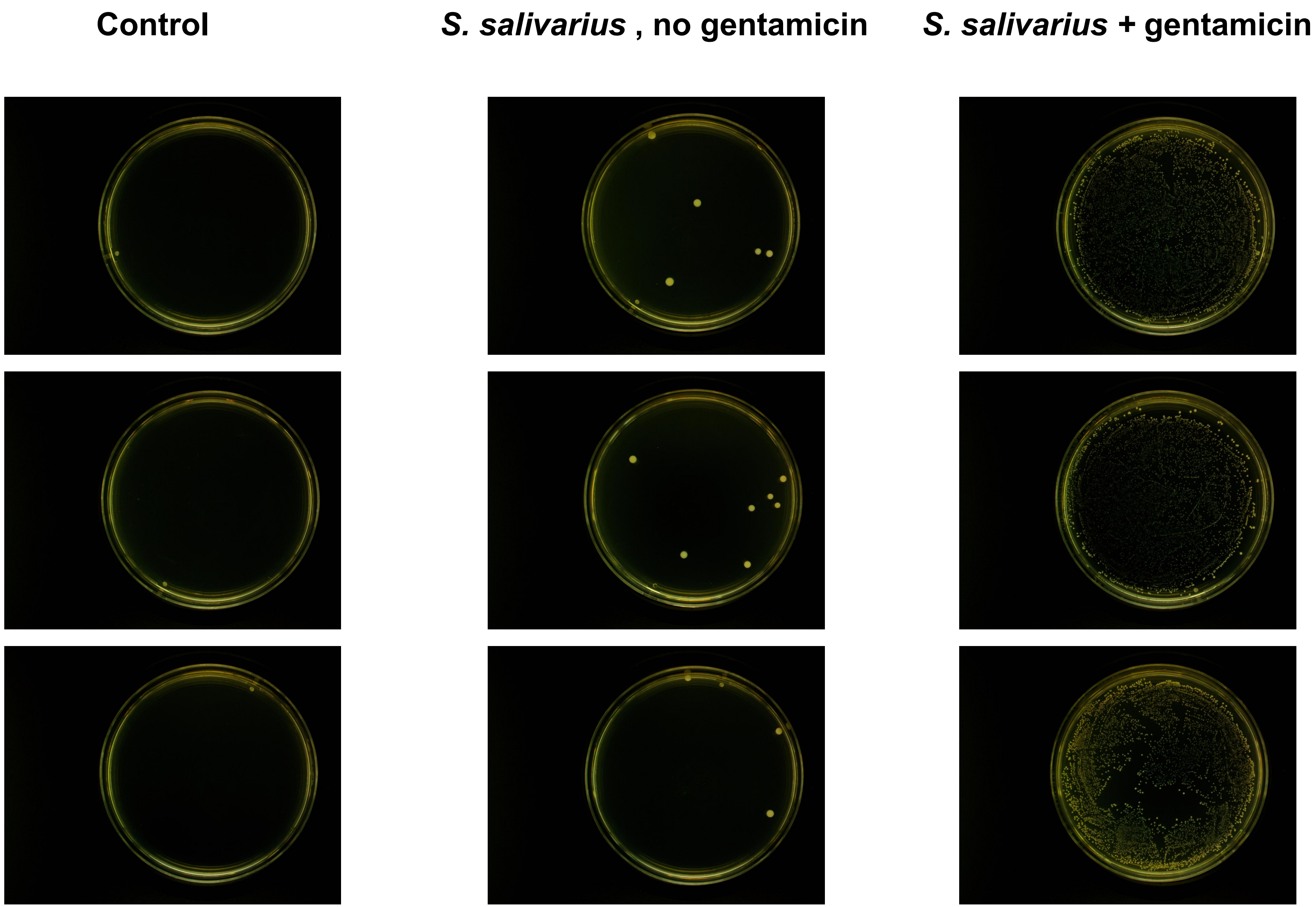

Figure S6. Gentamicin protection assay to assess the association of viable S. salivarius with HepG2-CYP2E1 cells.

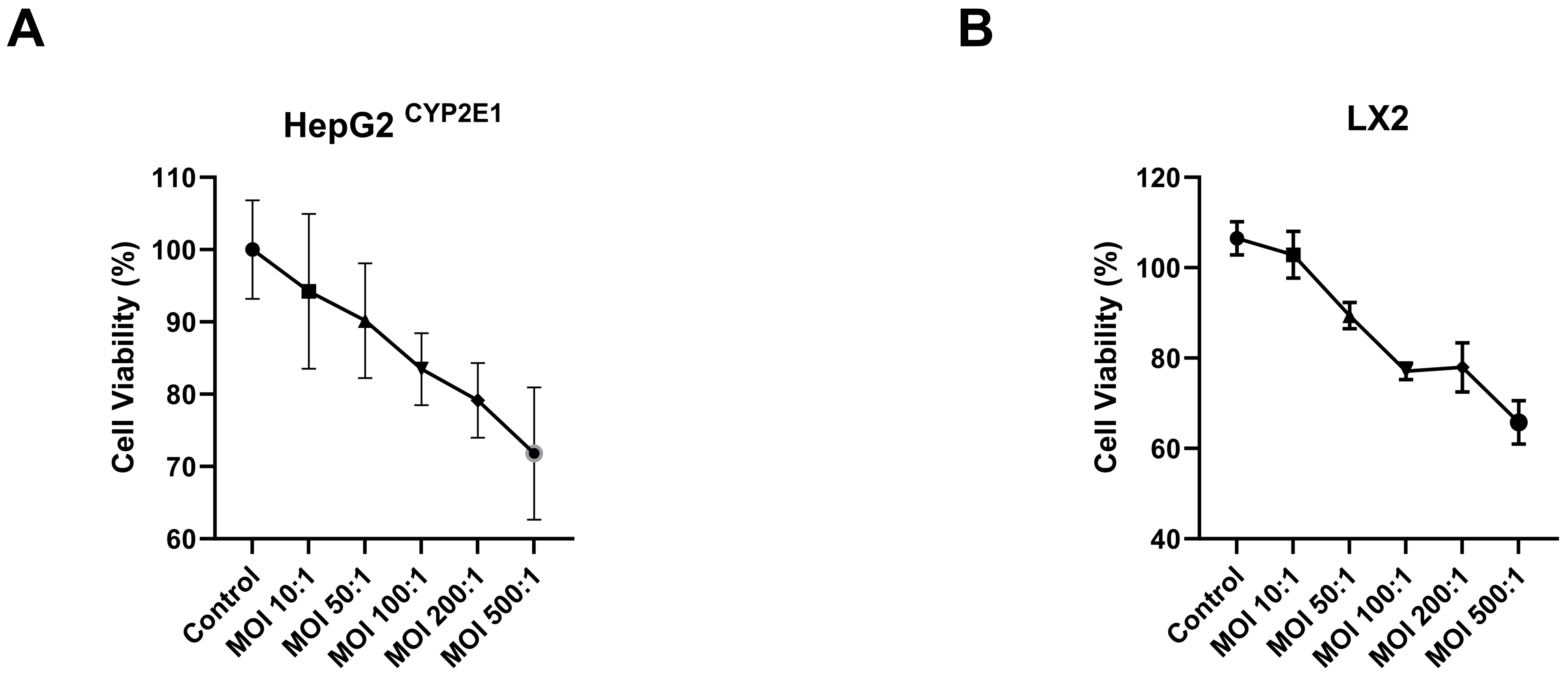

Figure S7. Effects of S. salivarius co-culture on the viability of HepG2-CYP2E1 and LX2 cells.

(A, B) HepG2-CYP2E1 cells (A) and LX2 cells (B) were co-cultured with logarithmic-phase S. salivarius at MOIs of 10:1, 50:1, 100:1, and 200:1 for 4 h.
